# Analysis of Sry duplications on the Rattus norvegicus Y-chromosome

**DOI:** 10.1186/1471-2164-14-792

**Published:** 2013-11-14

**Authors:** Jeremy W Prokop, Adam C Underwood, Monte E Turner, Nic Miller, Dawn Pietrzak, Sarah Scott, Chris Smith, Amy Milsted

**Affiliations:** 1 Department of Biology, The University of Akron, 302 Buchtel Commons, 44325-3908 Akron, OH, USA; 2 Program in Integrated Bioscience, The University of Akron, 44325 Akron, OH, USA; 3 Human and Molecular Genetics Center, Medical College of Wisconsin, 53226 Milwaukee, WI, USA; 4 Department of Mathematics and Science, Walsh University, North Canton, OH, 44720, USA

**Keywords:** Y-chromosome, Sry, SHR, *Rattus norvegicus*, Copy number variations, Y-chromosome evolution

## Abstract

**Background:**

Gene copy number variation plays a large role in the evolution of genomes. In *Rattus norvegicus* and other rodent species, the Y-chromosome has accumulated multiple copies of *Sry* loci. These copy number variations have been previously linked with changes in phenotype of animal models such as the spontaneously hypertensive rat (SHR). This study characterizes the Y-chromosome in the *Sry* region of *Rattus norvegicus*, while addressing functional variations seen in the Sry protein products.

**Results:**

Eleven *Sry* loci have been identified in the SHR with one (*nonHMG Sry*) containing a frame shift mutation. The *nonHMGSry* is found and conserved in the related WKY and SD rat strains. Three new, previously unidentified, *Sry* loci were identified in this study (*Sry3BII*, *Sry4* and *Sry4A*) in both SHR and WKY. Repetitive element analysis revealed numerous LINE-L1 elements at regions where conservation is lost among the *Sry* copies. In addition we have identified a retrotransposed copy of *Med14* originating from spliced mRNA, two autosomal genes (*Ccdc110* and *HMGB1*) and a normal mammalian Y-chromosome gene (*Zfy*) in the *Sry* region of the rat Y-chromosome. Translation of the sequences of each *Sry* gene reveals eight proteins with amino acid differences leading to changes in nuclear localization and promoter activation of a Sry-responsive gene. Sry-β (coded by the *Sry2* locus) has an increased cytoplasmic fraction due to alterations at amino acid 21. Sry-γ has altered gene regulation of the *Sry1* promoter due to changes at amino acid 76.

**Conclusions:**

The duplication of *Sry* on the *Rattus norvegicus* Y-chromosome has led to proteins with altered functional ability that may have been selected for functions in addition to testis determination. Additionally, several other genes not normally found on the Y-chromosome have duplicated new copies into the region around the *Sry* genes. These suggest a role of active transposable elements in the evolution of the mammalian Y-chromosome in species such as *Rattus norvegicus.*

## Background

The testis determining gene *Sry* on the mammalian Y-chromosome triggers the testis development pathway in placental mammals [1]. It encodes a protein that is composed of a highly conserved three helix HMG domain with additional hinge and bridge domains. Variations are found in the N- and C-terminal domains among species. In most mammals, *Sry* is found as a single locus; however, rodent species [2-5], have been reported to show copy number variation (CNV). CNV of *Sry* has been detected in humans exposed to radiation or those with sex chromosome related anomalies [6,7]. In *Rattus norvegicus,* multiple loci of *Sry* are expressed and code for proteins with altered amino acid sequences [8]. These loci have been suggested to function in the development of increased blood pressure in the Spontaneously Hypertensive Rat (SHR) compared to the normotensive Wistar Kyoto (WKY) [9]. One locus, *Sry3*, has been detected in SHR but not in WKY, and codes for a protein that has amino acid differences altering promoter regulation of renin-angiotensin system genes [10]. The entire rat Y-chromosome for *Rattus norvegicus* has yet to be assembled, thus limiting full understanding of gene duplication and divergence of *Sry* copies. Using newly deposited BAC clone sequences from the SHR rat strain (SHR/Akr), we have localized the *Sry* copies relative to each other, identifying several new *Sry* loci. The multiple copies of *Sry* code for proteins with amino acid variations resulting in functional changes to promoter activity and nuclear localization.

## Results

### Identification of Sry Copies in the Rattus norvegicus SHR strain

Several BAC sequences for the SHR/Akr Y chromosome have been deposited in Genbank. We have combined our past analyses of *Sry* in this strain with these new sequences, to identify contigs that include various *Sry* loci (Table 1). The current number of identified *Sry* loci in *Rattus norvegicus* is eleven*.* The novel eleventh locus we have designated as *nonHMG Sry* due to a frame shift mutation yielding a protein with an incomplete HMG box.

**Table 1:** **BAC sequences used to assemble the *Sry *** **region of the SHR Y-chromosome**

**BAC**	***Sry*** **gene(s)**	**BAC status**	**Library**	**Fragments**	**Bases**	**Date deposited**
AC242859	*Sry3A*	Complete	RNAEX	1	99871	Dec 2010
AC239701	*Sry4*	Complete	RNAEX	1	138396	Dec 2010
AC239865	*nonHMG, Sry4A*	Complete	RNAEX	1	268785	Dec 2010
AC239814.3	*Sry4*	working draft	RNAEX	1	331097	Sept 2011
AC239817	*Sry3B*	Complete	RNAEX	1	97422	Dec 2010
AC239866.3	*nonHMG*	working draft	RNAEX	23	92289	Feb 2010
AC243747.5	*Sry3C, Sry3B*	working draft	RNECO	7	165825	Apr 2011
AC243673	*Sry3C Sry4A*	working draft	RNECO	13	153940	Apr 2011
AC243442	*Sry4A, Sry3C, Sry1*	working draft	RNECO	11	100422	Dec 2010
AC242641	*Sry3A, Sry3BI*	working draft	RNECO	6	144578	Sep 2010
AC241808.7	*Sry3B, Sry3BII, Sry2*	Complete	RNECO	1	156555	Jun 2011
AC240535	*Sry3BII, Sry2*	Complete	RNECO	1	153894	Dec 2010

**Figure 1:**
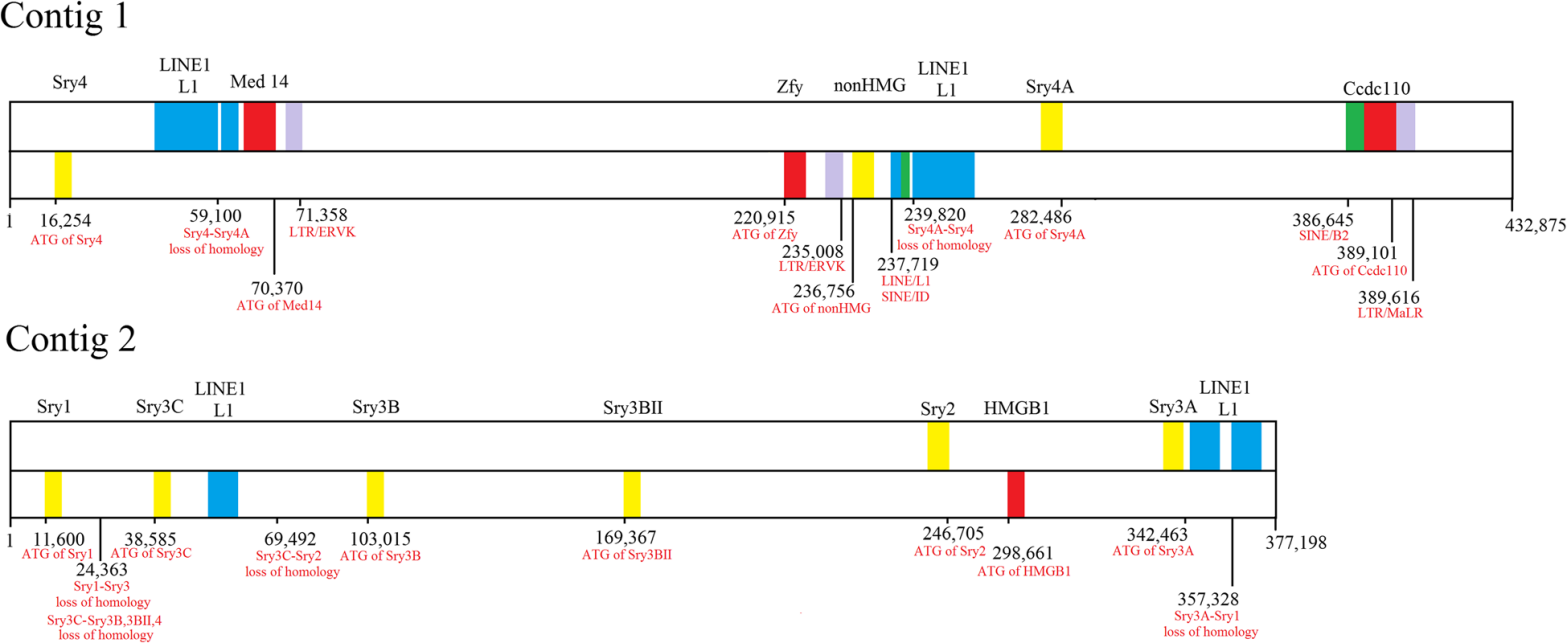
**Contigs containing the multiple *Sry *genes of the SHR Y-chromosome.***Sry* genes are shown in yellow, other genes (*Med14*, *Zfy*, *Ccdc110*, and *HMGB1*) in red, LINE-1 elements in cyan, LTR elements in purple, and SINE elements in green. Numbering is given for major positions (such as the ATG start codons and loss of homology between copies) in red relative to the beginning of each contig.

The assembly of the *Sry* region has resulted in two aligned contigs of 432 kb and 377 kb, respectively. Using these contigs, the location and identity of multiple *Sry* loci were mapped (Figure 1). The first contig (Contig 1) contains *Sry4*, *nonHMG Sry* and *Sry4A* while the second (Contig 2) contains *Sry1*, *Sry3C*, *Sry3B*, *Sry3BII*, *Sry2*, and *Sry3A*. In addition to the *Sry* loci found in these two contigs, two additional previously identified *Sry* loci (*Sry3* and *Sry3BI*) did not map to either contig. Repeatmasker identified a high level of LINE L1 elements in both Contig 1 (31.38%) and Contig 2 (32.77%) relative to DNA sequence flanking the single copy of human *SRY* (*hSRY*, 18.88%) or mouse *Sry* (*mSry*, 17.70%,) (Additional file 1: Figures S1-S4). Alignment of sequences for the multiple rat *Sry* loci reveals regions conserved among the various copies (Additional file 1: Figure S5). Many of the sites where homology is lost are flanked by LINE L1 elements (blue, Figure 1). Phylogenetic analysis using the sequence conserved in *Sry* copies in these contigs shows clustering of the *Sry3* loci (*3BII, 3A, 3B, 3C*) and a separate clustering of *Sry4* and *Sry4A* (Additional file 1: Figure S6).

### Newly identified Sry copies in other Rattus norvegicus strains

Four novel *Sry* sequences (*nonHMG Sry, Sry4*, *Sry4A* and *Sry3BII)* were identified in the SHR/Akr in this paper. Of these, the *nonHMG Sry* sequence was confirmed from genomic DNA, with 100% sequence homology in SHR/Akr [GenBank: KC215139], WKY/Akr [KC215140], and SD/hsd [KC215141] strains. *Sry4*, *Sry4A* and *Sry3BII* were confirmed in both SHR/Akr and WKY/Akr strains using PCR reactions and selective restriction digests designed to differentiate loci based on PCR products (Additional file 1: Figure S7).

### Identification of other loci in the Sry region of the SHR Y-chromosome

The *zinc finger on the Y* (*Zfy*) locus is found between *Sry4* and the *nonHMG Sry*. This locus is found on many other mammalian Y-chromosomes near *Sry*. In addition to *Zfy*, we have identified a possible *Med14* locus between *Sry4* and the *nonHMG Sry*. Normally *Med14* is found on the mammalian X-chromosome, where it contains 31 exons separated by large introns. The form identified on the Y-chromosome of SHR is spliced, containing 30 of the normal 31 exons of the long form *Med14* of the X-chromosome (Additional file 1: Figure S8). Located 1kB from the *Med14Y* sequence (red, Figure 1) is an LTR/ERVK element (purple, Figure 1). The presence of both an LTR element and spliced exons suggests that this copy has been retrotransposed onto the Y-chromosome. The protein coded by *Med14Y* would be highly homologous to the X-chromosome *Med14* (Additional file 1: Figure S9) if it could be transcribed and translated. In addition to *Med14Y*, we have identified a *Ccdc110-like* locus (Contig 1, after *Sry4A*) and a *HMGB1-like* locus (Contig 2, between *Sry2* and *Sry3A*, Figure 1).

### Sry protein sequence comparison

**Figure 2:**
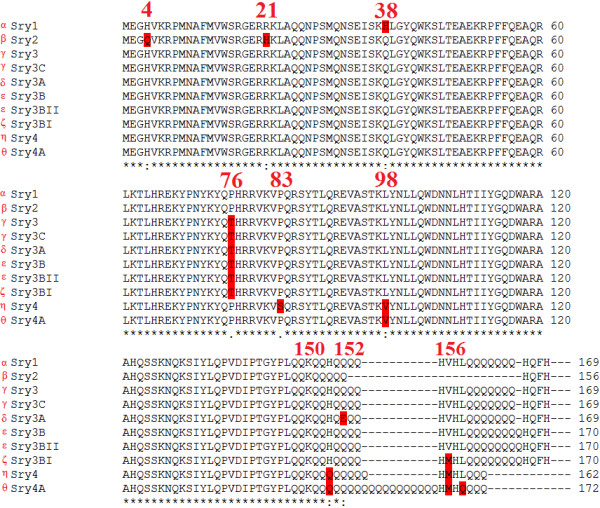
**Sequence alignments of the proteins coded for by the multiple *Sry *loci.** The Greek symbol for each Sry protein is included to the left of each gene name. Amino acid variations are highlighted in red with the rat protein numbering system used to identify the location of these variations.

**Figure 3:**
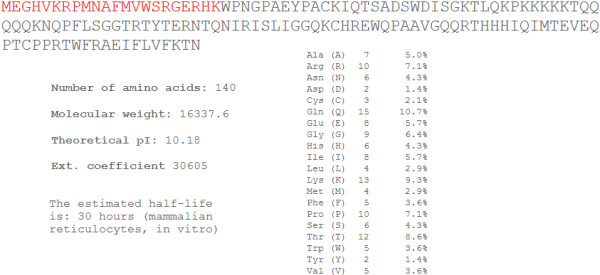
**Sequence of the nonHMG Sry with ProtParam statistics.** Sequence shows the conserved first helix of the HMG box in red, followed by a frame shift mutation leading to a different protein sequence. The predictions for molecular weight, pI, and extinction coefficient are given. This protein sequence contains a high percentage of Gln (10.7%) and Lys (9.3%) as can be seen in the amino acid percentages.

Translating each of the *Sry* loci into protein sequences (Table 2) allows for comparison of amino acid variations. The eleven *Sry* loci can be translated into nine (Sryα-Sryθ and nonHMG Sry) different protein sequences. Several *Sry* loci encode the same protein sequence; therefore, we created a new naming system for the Sry proteins using Greek letters to help to differentiate protein naming from the *Sry* loci naming. The *Sry3*/*Sry3C* (Sryγ used to symbolize the protein) and *Sry3B/Sry3BII* (Sryϵ) loci translate to the same protein sequences (Figure 2). Sequence alignments of all Sry proteins reveal variation at amino acids 4, 21, 38, 76, 83, 98, 150, 152, 156 and in the Q-repeat length of the C-terminus. The *nonHMG Sry* codes for a highly divergent sequence containing the first helix and nuclear localization site of normal Sry (red, Figure 3), but due to a frame shift mutation the sequence diverges after this region. This protein sequence following the frame shift mutation contains a high level of Lys and Gln amino acids (Figure 3), indicating a high potential for interactions with other proteins.

**Table 2:** Sry proteins encoded on the SHR Y-chromosome with GenBank accession codes

**Sry gene**	**Sequenced BACs (s)**	**Protein product**	**Confirmed sequence in SHR/Akr**	**Confirmed sequence in WKY/Akr**
*nonHMG Sry*	AC239865.4	Predicted	KC215139	KC215140
*Sry1*	AC243442	α	EU984075	FJ168067
*Sry2*	AC240535.5	β	FJ168057	FJ168068
*Sry3*	Not yet identified in BACs	γ	EU984077	Unconfirmed
*Sry3A*	AC242859.2	δ	EU984078	FJ168069
*Sry3B*	AC241808.7, AC239817.2	ϵ	FJ168058	FJ168070
*Sry3BI*	AC242641	ζ	FJ168059	FJ168071
*Sry3BII*	AC240535.5, AC241808.7	ϵ	Present	Present
*Sry3C*	AC243442	γ	EU984076	FJ168072
*Sry4*	AC239701.6	η	Present	Present
*Sry4A*	AC239865.4	θ	Present	Present

**Figure 4:**
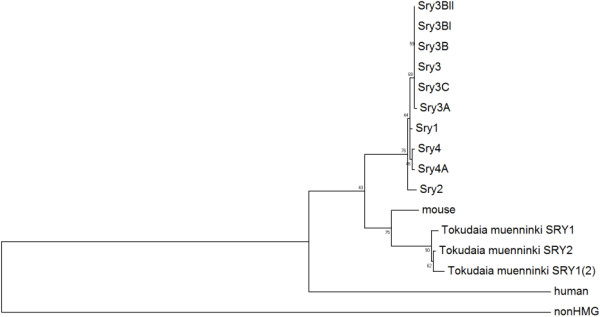
**Phylogenetic analysis of the coding region of the multiple rat Sry.** Phylogenetics of the coding region of the rat Sry genes compared to the mouse Sry, three copies of *Tokudaia muenninki*, human and the nonHMG. Numbers next to each node represent the percent of the 500 bootstrap phylogenies that were identified similar to the one shown in the figure.

Using the DNA sequence, starting at the ATG start codon and extending through the stop codon of each *Sry* copy, phylogenetic analysis was performed comparing rat *Sry* copies to Muennink’s Spiny rat (*Tokudaia muenninki*) copies, *mSry*, and *hSRY* (Figure 4). All the proteins coded by the *Sry3* loci (Sryγ-Sryζ) cluster together (as also confirmed through conserved amino acids), as do those from *Sry4* and *Sry4A* (Sryη-Sryθ), similar to the analysis done using the maximally conserved sequences (Additional file 1: Figure S6). The phylogenetic analysis suggests the possibility that a *Sry* locus, present before the *Sry2* split from *Sry1*, *Sry4* and *Sry4A,* may have been the original locus in rodents prior to copy number increase. Inspection of individual amino acids found in the rat *Sry* copies when compared to human and mouse protein sequences supports this claim (Figure 2 and Additional file 1: Figure S10). As expected, the *nonHMG Sry* sequence is highly divergent from all other *Sry*, a likely result of drift following the frame shift mutation.

### Sry protein functional variations

**Figure 5:**
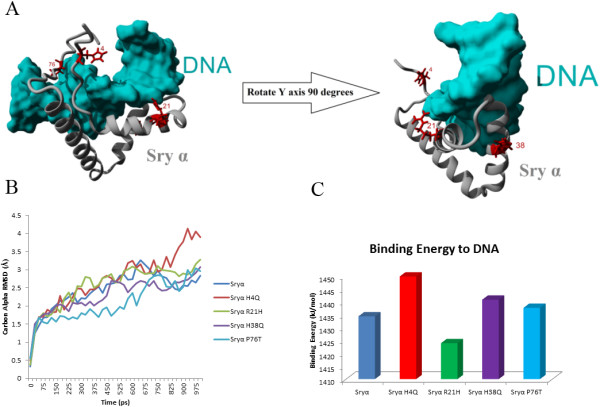
**HMG box variations in the rat Sry proteins. A)** Amino acid variations (red) found in the HMG box of rat Sry proteins shown on a model of Sry1 (gray) when bound to DNA (cyan) based on the known human structure. **B)** Averaged movement during molecular dynamic simulations for models of Sryα interacting with DNA with each mutation from A. Shown as the averaged carbon alpha RMSD for the structure over a 1 nanosecond simulation. **C)** Predicted binding energy to DNA of the energy minimized structures of Sryα or containing one of the four mutations in Sryα.

To analyze functional variation of amino acid differences in the multiple Sry proteins, this study used both modeling and site directed mutagenesis. Three amino acid sites differ in the HMG box (amino acids 4, 21 and 38; Figure 2 and Additional file 1: Figure S10) and an additional amino acid site in the highly conserved hinge domain (amino acid 76; Figure 2 and Additional file 1: Figure S10). Amino acids 4, 21, and 76 additionally fall into nuclear localization signal sites [11,12]. Using a modeled structure of the protein coded by the *Sry1* locus (Sryα) interacting with DNA (cyan, Figure 5A), it can be seen that substitutions in amino acids 4, 21 and 76 (red, Figure 5A) may have a slight propensity to alter DNA binding stability (Figure 5B) and energy (Figure 5C and Additional file 1: Figure S11).

**Figure 6:**
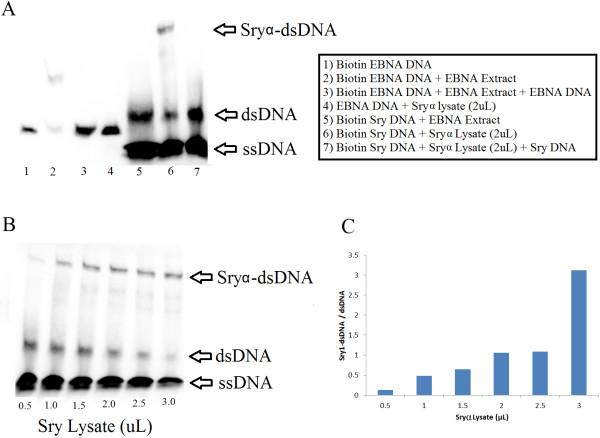
**Binding of Sryα to a known Sry-DNA binding element. A)** Control experiments for Sryα specific binding. The protein and DNA identities for each lane can be seen in the box next to the gel. **B)** Concentration curve for extract amounts of *Sry1* transformed *E. coli* cell lysates expressing Sryα protein. **C)** Densitometry measurements of the Sryα-dsDNA and dsDNA bands mapped relative to lysate volume.

Sryα binds to DNA elements that are known to be bound by hSRY in a sequence specific manner (Figure 6A). Use of a control EBNA DNA (lane 1) is shifted by EBNA protein extract (lane 2), with the shift outcompeted with unlabeled DNA (lane 3). Adding Sryα lysate to this EBNA DNA does not shift the band (lane 4), confirming specificity of DNA sequence for binding. Taking a Sry DNA binding element and adding the EBNA protein did not result in a shift (lane 5) while addition of Sryα lysate did (lane 6). The Sryα binding is competed off with unlabeled Sry DNA (lane 7). The shifting of the DNA was concentration dependent on Sryα lysate (Figure 6B-C). This confirms for the first time that Sry protein from *Rattus norvegicus* can bind to similar DNA sequences as human and mouse.

**Figure 7:**
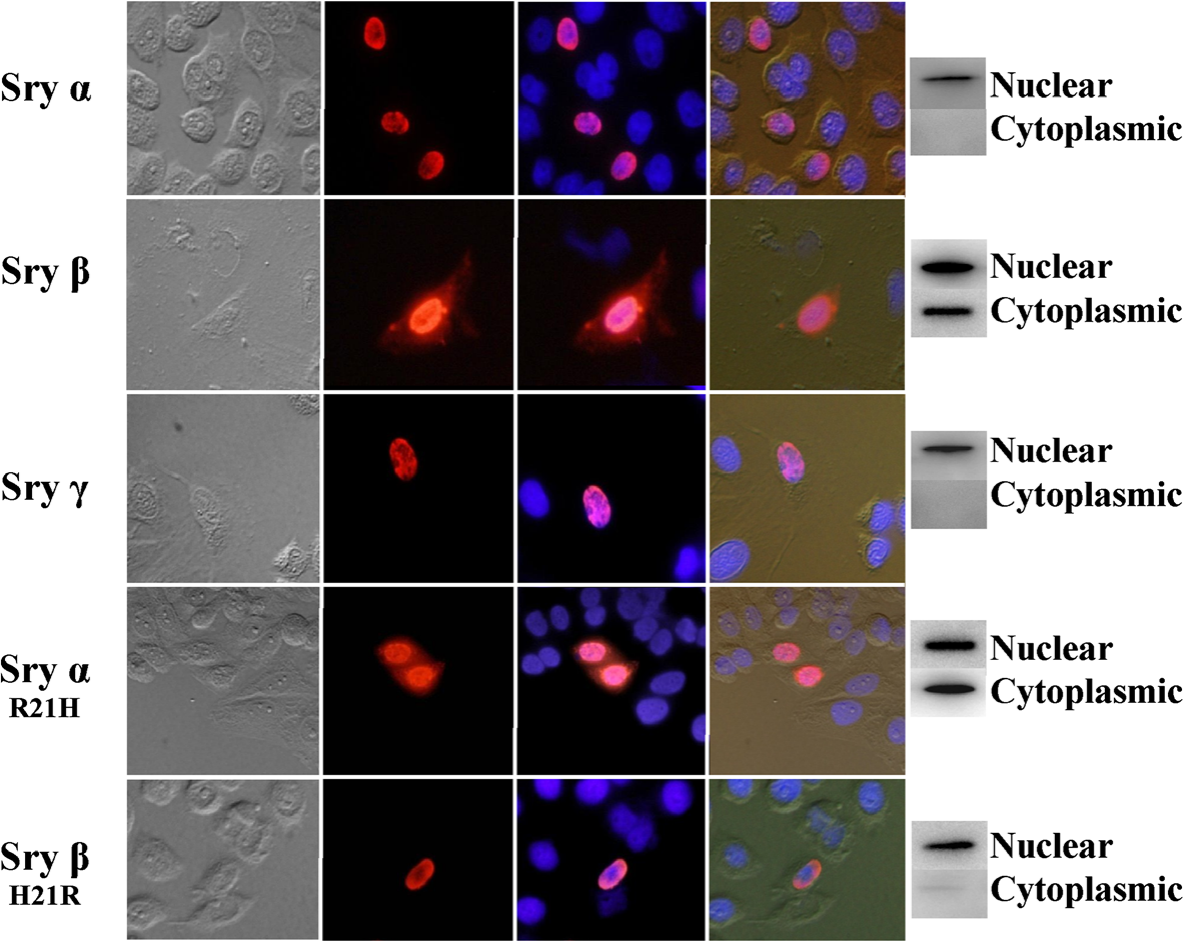
**Immunocytochemical and Western blot analysis of native and mutated Sry protein cellular distribution.** Cells were transfected with Sryα (top), Sryβ, Sryγ, Sryα R21H, or Sryβ H21R (bottom) and analyzed with microscopy and western blot for cellular distribution. The first (left most) column of images shows the transfected CHO cells visualized with light microscopy, the second with Cy3 (red) detection of the respective Sry (with Cy3 conjugated to the secondary antibody recognizing the Sry specific antibody), third the DAPI staining (blue, recognizing the nucleus) with Cy3 overlay, and the fourth (right most column) the overlay of all columns allowing for identification of the nucleus and cell outline together with Sry expression (red). To the right of the images of the cells are western blots of the nuclear and cytoplasmic fractions from CHO cell lysates. Sryα and Sryγ proteins localize exclusively to the nucleus while Sry β is distributed in both the nucleus and cytoplasm. Chimeric Sry β(H21R) encodes an R at residue 21, as seen in Sryα/γ, rather than H, while Sry α(R21H) proteins contain a H residue at position 21 as see in native Sryβ. The resulting staining patterns demonstrate that an R encoded at position 21 stimulates nuclear localize while a H at this position as see in native Sry β and chimeric Sryα (R21H) results in cytoplasmic accumulation. Western blots of nuclear and cytoplasmic extractions support the results from the immunocytochemical analysis.

Sryα and Sryγ primarily localize to the nucleus of the cell, while Sryβ is also found in the cytoplasm (Figure 7). Mutating amino acid 21 in Sryα from an Arg to a His (Sryα R21H) resulted in more Sry localization in the cytoplasm, while mutating amino acid 21 in Sryβ from His to Arg (Sryβ H21R) abolished the cytoplasmic localization. This confirms that changes to amino acid 21 can alter cellular localization, while changes at amino acid 38 and 76 likely have minimal change to cellular localization (comparing Sryα and Sryγ).

**Figure 8:**
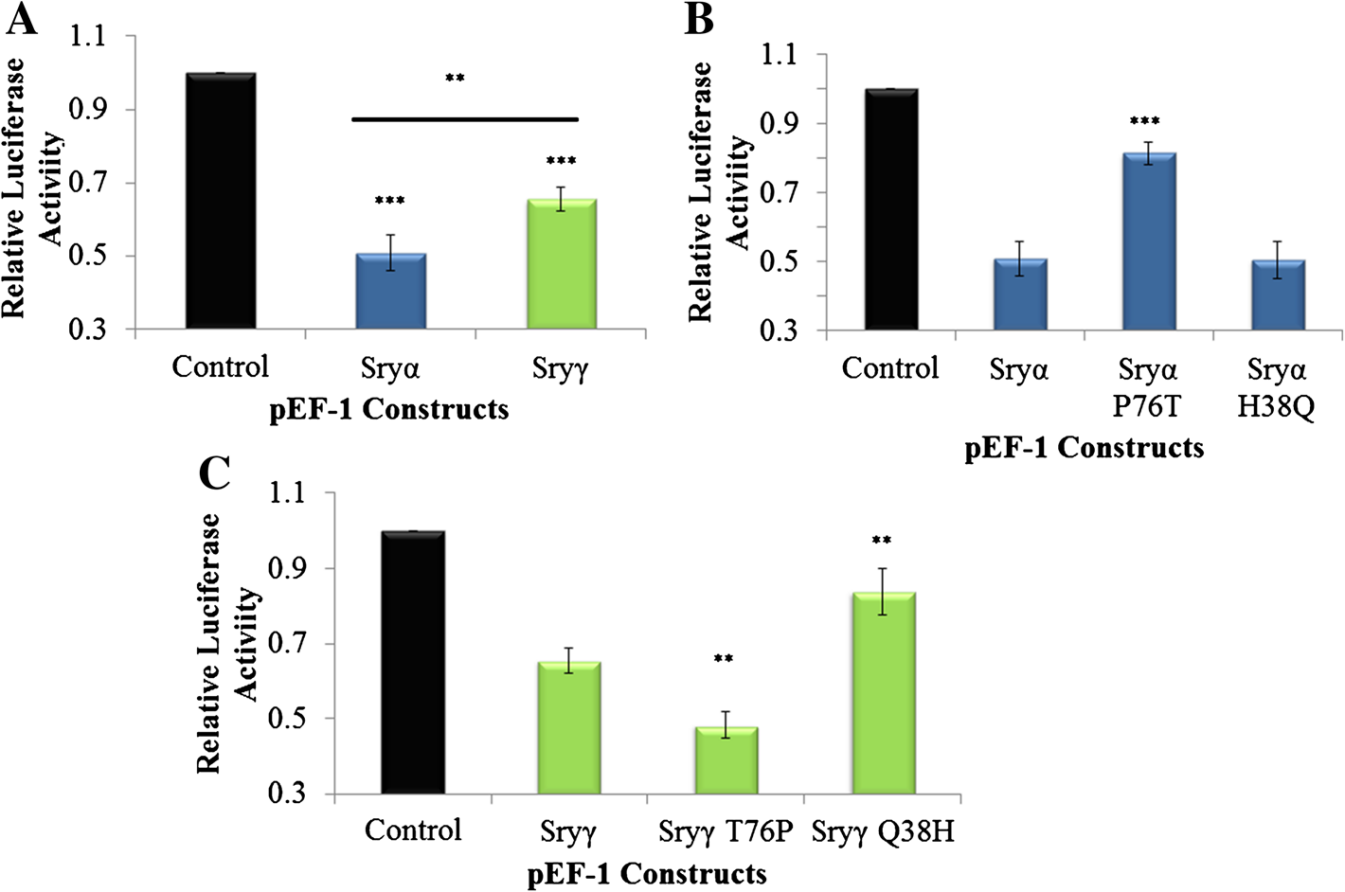
**Regulation of the *Sry1 *promoter by Sryα and Sryγ. A)** Sryα (blue) and Sryγ (green) transfected CHO cells significantly repress the *Sry1* promoter construct when compared to empty vector control (black) or each other (bar above Sryα and Sryγ). **B)** Sryα and mutations of Sryα at amino acids 76 (Sryα P76T) and 38 (Sryα H38Q) transfected into CHO cells all repress the *Sry1* promoter construct when compared to empty vector control (black), with the P76T and not H38Q significantly differing from Sryα. **C)** Sryγ and mutations of Sryγ at amino acids 76 (Sryγ T76P) and 38 (Sryγ Q38H) transfected into CHO cells all repress the *Sry1* promoter construct when compared to empty vector control (black), with the T76P and Q38H significantly differing from Sryγ. For all figures error bars represent the standard error of the mean and statistical significance is shown as *∗ = p ≤* 0*.*05, *∗∗ = p ≤* 0*.*01, *∗∗∗ = p ≤* 0*.*001.

The presence of Thr at amino acid 76 defines the *Sry3* subgroup (Sryγ-Sryζ, Figure 2). Sry in all other mammalian species (340 species analyzed to date), every member of the Sox subfamily to which Sry belongs, and rat Sryα/β/η/θ proteins all contain a Pro at amino acid 76. Comparing Sryα to Sryγ, only two amino acids vary (38 and 76). The *Sry1* promoter is differentially repressed by Sryα and Sryγ compared to the control or each other (Figure 8A). When amino acid 76 is mutated in Sryα from a Pro to a Thr, this decreases the *Sry1* promoter regulation, yielding less repression relative to Sryα (Figure 8B). Changing amino acid 76 in Sryγ from a Thr to a Pro increased *Sry1* promoter repression relative to Sryγ (Figure 8C). Changing amino acid 38 from His to Gln in Sryα had no effect on the promoter regulation (Figure 8B). In contrast, when amino acid 38 of Sryγ was changed from Gln to His, promoter repression was decreased relative to Sryγ (Figure 8C).

**Figure 9:**
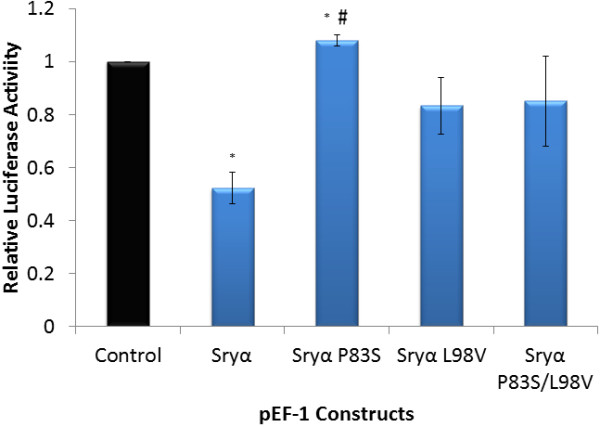
**Activity of the *Sry1 *promoter with amino acid changes found in Sryα and Sryη/θ.** Sryα and Sryα mutated at sites corresponding to Sryη/θ amino acid differences (P83S, L98V, and P83S/L98V double mutant) transfected into CHO cells and regulating the *Sry1* promoter construct when compared to empty vector control (black). Error bars represent the standard error of the mean and * = p *≤* 0*.*05 comparing each value to the control while # = p *≤* 0*.*05 comparing Sryα and Sryα P83S.

Comparing the proteins from the *Sry4*/*4A* loci (Sryη/θ proteins) with those of the previously identified Sry proteins, amino acids 4, 21, and 38 are conserved with Sry γ-ζ (the *Sry3* loci), while amino acid 76 is shared with Sryα, Sryβ and all other known mammalian Sry proteins (Figure 2 and Additional file 1: Figure S10). Two unique amino acid variations from the rest of the rat Sry proteins are found in the bridge domain of Sryη/θ. Mutations of Sryα at amino acid 98 (from a Leu to Val designated as Sryα L98V, similar to Sryθ) showed a trend towards decreased repression of the *Sry1* promoter relative to Sryα (Figure 9). A double mutant (similar to Sryη) at amino acid 98 and also 83 (Pro to Ser; Sryα P83S/L98V) showed regulation similar to the single mutant construct (Sryα L98V). Sryα P83S caused a significant loss of regulation on the *Sry1* promoter construct; however, this variant is not found by itself in any of the rat Sry copies.

**Figure 10:**
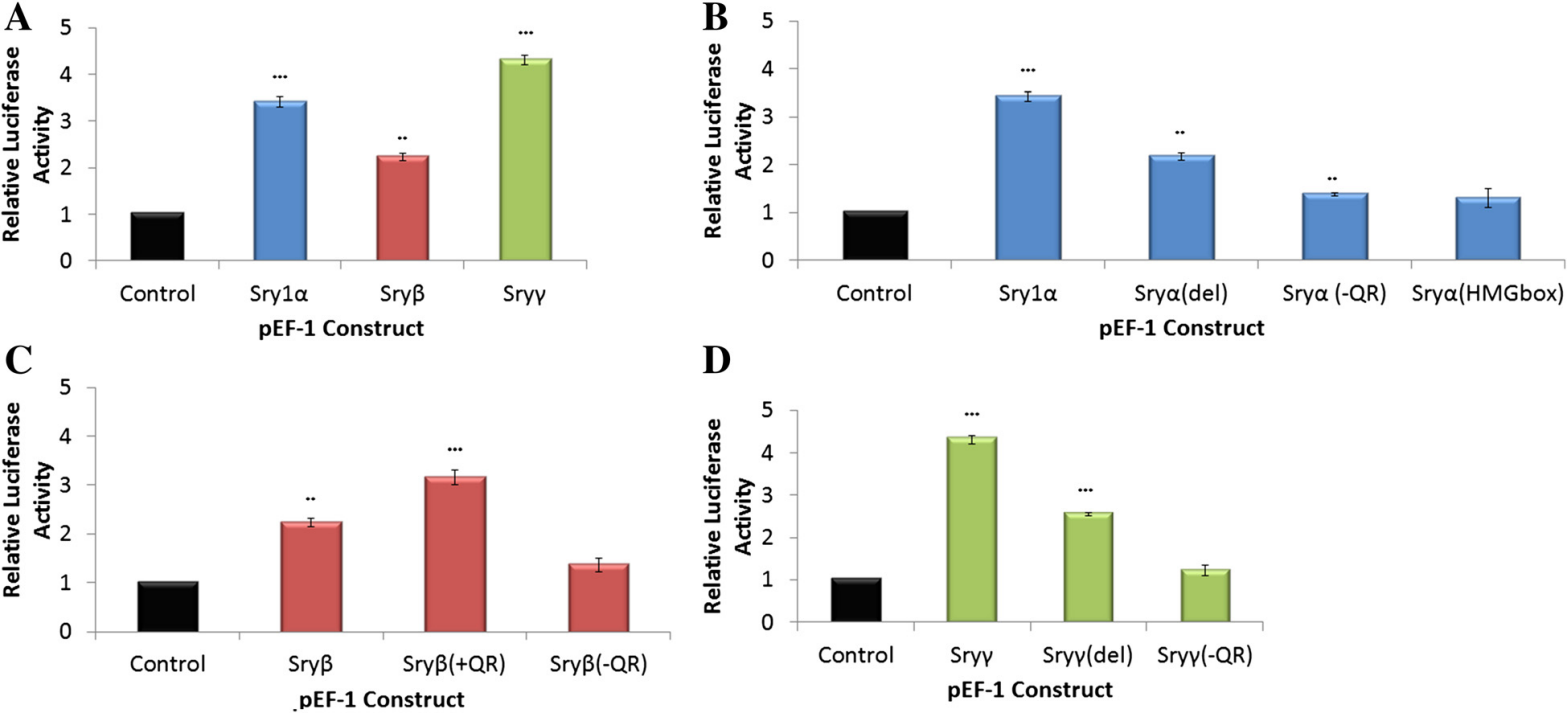
**Function of the Q rich region of the C-terminal end of Sry. A)** Regulation of the AR600 promoter construct by Sryα (blue), Sryβ (red) and Sryγ (green). Multiple mutations made to Sryα **(B)**, Sryβ **(C)** or Sryγ **(D)** that altered AR600 promoter regulation. For Sryα three deletion constructs were created **(B)**: Sryα(del) removing 13 amino acids in the Q-rich region making it more similar to Sryβ, Sryα(−QR) removing the whole 25 Q-rich region, and Sryα(HMGbox) containing only the HMG box of Sryα . For Sryβ two alternative constructs were created **(C)**: Sryβ(+QR) adding the 13 amino acids of Sryα in the Q-rich region missing from Sryβ, Sryβ(−QR) removing the entire 25 amino acids of the Q-rich region. Two alternative constructs were created for Sryγ **(D)**: Sryγ(del) removing 13 amino acids in the Q-rich region making it more similar to Sryβ, Sryγ(−QR) removing the whole 25 Q-rich region. Error bars represent the standard error of the mean and significance is shown as *∗ = p ≤* 0*.*05, *∗∗ = p ≤* 0*.*01, *∗∗∗ = p ≤* 0*.*001 for each compared to the control (black).

**Figure 11:**
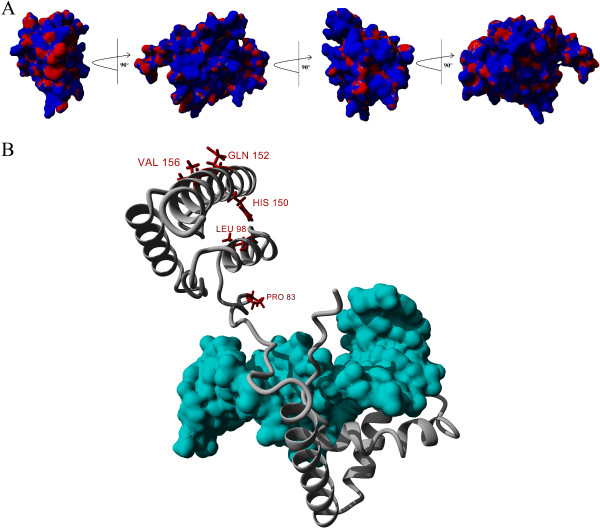
**Structural model of full-length rat Sryα protein. A)** The modeled C-terminal end containing the Q-rich region of Sryα showing the electrostatic surface as determined with Poisson-Boltzmann Solver (blue is positive charge and red is negative charge). The model was rotated 90° on the Y-axis for four views yielding a 360° structural analysis. The model had a quality score (z-score) of −1.564 as determined with the YASARA2 force field (anything greater than −2 is considered a fair model), and contains no wrong isomers or cis-peptide bonds. **B)** Ribbon view of the structure of the C-terminal segment (as seen in the left most image in **A)** added to the HMG box of Sryα bound to DNA (cyan). Amino acid variations between the multiple rat *Sry* loci falling outside the HMG box are shown in red with the rat numbering system used (Figure 2).

Sry proteins with different lengths of the Q-rich C-terminal region (Sryα, Sryβ and Sryγ, Figure 2) activate the *AR600* promoter construct at various levels compared to the control (Figure 10A). Sryβ has a deletion of 13 amino acids when compared to Sryα and Sryγ (Figure 2). Removing these 13 amino acids from Sryα, creating a construct known as Sryα(del), decreased promoter activity compared to the wild type Sryα (Figure 10B) yielding similar values as Sryβ. Completely removing the 25 amino acids of the Q-rich region of Sryα, known as Sryα(−QR), resulted in minimal regulation of the promoter as did a construct containing only the HMG box of Sryα (Figure 10B). Sryβ with the 13 Q amino acids of Sryα or Sryγ added, known as Sryβ (+QR), increased promoter activity compared to Sryβ, yielding a value similar to Sryα and Sryγ (Figure 10C). The complete removal of the Q-rich region of Sryβ, Sryβ (−QR), caused a complete loss of promoter regulation. Similar results to mutations of Sryα (Figure 10B) were seen when the deletions were made to Sryγ (Figure 9D). Modeling the Q-rich region and the remaining C-terminal end of rat Sry (Figure 11A) shows the locations of variations outside of the HMG box mapped onto the structure of Sryα interacting with DNA (Figure 11B, Additional file 2).

## Discussion

Multiple *Sry* loci in *Rattus norvegicus* are known to be expressed [8]. Sequencing of the SHR/Akr rat Y chromosome is under progress by the Y chromosome project (http://www.genome.gov/25521746). Identifying and labeling all the *Sry* loci and functional variations are of importance for both annotating Y-chromosome sequence data and for understanding how each of these *Sry* loci functions. The SHR/Akr strain has been shown to have loci on the Y-chromosome that are associated with blood pressure increases [13,14] and other sympathetic nervous system phenotypes reviewed in [15]. Animals with the Y-chromosome of SHR and WKY autosomes have higher blood pressure than WKY males [14]. Studies have revealed the inability to amplify the *Sry3* locus, present in SHR, from WKY genomic DNA, suggesting a potential increase in CNV of SHR over WKY [9]. Delivery of the coding sequence of either *Sry1*[16] or *Sry3*[17] but not *Sry2*[16] into the kidney of normotensive WKY rats induces a blood pressure increase through the renin-angiotensin system and the sympathetic nervous system. With *Sry3* present in SHR but not detected in WKY [10], this additional copy of an *Sry* gene (*Sry3*) may be largely responsible for the blood pressure elevation seen in Y-chromosome crosses of the consomic strains [13,14]. Y-chromosome sequence analysis has revealed at least eleven *Sry* loci on the Y-chromosome of *Rattus norvegicus* SHR/Akr that code for nine different proteins. It is possible that additional loci exist and have yet to be sequenced; sequencing of the SHR/Akr Y-chromosome is still underway. We do not know which *Sry* locus codes for testis determination in *Rattus norvegicus* or if multiple loci contribute to this function. It may be possible that any of the ten *Sry* loci (excluding the *nonHMG Sry*) could determine sex if expressed with proper developmental timing. Four new loci were detected in this current study. These were previously unidentified due to an insertion of DNA after the 3’ untranslated region in these newly identified loci, preventing PCR amplification with the primer sets previously used. New primer sets and restriction digests allowed us to identify these new loci in both SHR and WKY rats.

Compiled contigs for the Y-chromosome region containing the *Sry* loci in *Rattus norvegicus* allow for identification of possible mechanisms of gene duplications. Of the four loci identified in our contigs that are not *Sry* (*Zfy*, *Med14*, *Ccdc110*, and *HMGB1*), several are flanked by repetitive elements that may have contributed to locus positioning on the Y-chromosome. An LTR/ERVK element was identified 5’ of the start codon of the *Med14Y*. This *Med14Y* is highly homologous to the X-chromosome *Med14*, however it is missing the normal introns, suggesting retrotransposition of a mature mRNA onto the Y-chromosome of rat. An LTR element has also been identified 3’ of the *Zfy* and 5’ of the *Ccdc110* loci. Currently we do not know the function or conservation (presence and spatial organization) of these loci on the Y-chromosome in other rat species. Future work will be required to determine the dynamic gene evolution occurring in rat Y-chromosome evolution.

The largest conserved fragment of DNA sequence within the contigs is between *Sry4* and *Sry4A*, with a large region of 3’ conservation (around 43 kB). Analysis of the conservation revealed a LINE L1 element (cyan, Figure 1) conserved at the point where homology is lost; this LINE L1 appears to have the proper sequences to code for proteins of both ORFs required for LINE L1 transposition. Flanking several other loci (*Sry3C* and *Sry3A*) are insertions of LINE L1 elements relative to the other loci, suggesting more recent LINE L1 insertions after copy number increased. SOX genes have been shown to regulate the promoters of LINE-L1 elements [18] suggesting a potential interplay between Sry and LINE-L1. These elements, as well as sequence variations, may alter the expression of the various loci, as has already been shown [8]. The *Sry2* gene is preferentially expressed in adult adrenal glands relative to testis, while the combination of *Sry1*, *Sry3* and *Sry3C* is expressed at a higher level in the testis relative to adrenal. The *Sry3C* locus shares a high level of conservation with the 3’ end of *Sry2* and a lower level with *Sry1*. These observations suggest that *Sry3C* may have resulted from the original duplication event and then further duplicated and differentiated to give the remaining *Sry3* subgroup members. Identifying the mechanism of duplication and order of events is complicated by a continual change to all loci after the duplication events occurred. This may be simplified by understanding the organization of the Y-chromosome and Sry sequences in other *Rattus norvegicus* strains and other rodent species. For example, our initial sequence analysis comparing the *Rattus norvegicus* and *Tokudaia muenninki Sry* copies did not reveal any connections between the duplicated copies at either the DNA or amino acid level, indicating that *Sry* duplication has occurred more than once. Understanding the duplication events in the rat may help to explain duplications of *SRY* seen in humans exposed to radiation or with Turner syndrome [6,7].

Amino acid differences among the proteins coded by the multiple *Sry* loci result in functional variation. From the eleven loci, we predict nine protein sequences (Sryα-Sryθ and the nonHMGSry). Sryβ has several amino acid variations in nuclear localization sites (amino acids 4 and 21) and also the C-terminal end (Figure 2 and Figure 11). Sry protein localizes to the nucleus using two different localization signals (NLS) [19]. The first NLS is found as a bipartite signal known to interact with calmodulin [20-22]. Amino acid 4 falls into the NLS in the first of the bipartite sequences and amino acid 21 in the second. Mutations of amino acid 21 in Sryα decreased nuclear localization, and models for the interaction between Sry and calmodulin suggest a decreased interaction when amino acid 21 is a His (Additional file 1: Figure S12).

All the *Sry3* loci (Sryγ-ζ proteins) contain a threonine at amino acid 76, rather than the normal proline. A proline is found at this amino acid in all human *SOX* genes (and all other vertebrate and invertebrate *Sox* genes analyzed to date by our lab) and all mammalian *Sry* sequences [23]. Variation at amino acid 76 in hSRY is associated with sex reversal [24]. This suggests a high degree of conservation, likely required for proper DNA bend angle, protein recruitment, and gene regulation [20]. Amino acid 76 falls in the second NLS which is thought to work through importin-β1 [25]. Both Sryα and Sryγ localize to the nucleus (Figure 7), and changes from a proline to a threonine at amino acid 76 do not seem to alter nuclear localization. Promoter activity assays show that the proline to threonine change in Sryα leads to changes in activity. Modeling approaches and proline to threonine mutations also showed altered regulation of renin-angiotensin system promoters [10] in addition to the *Sry1* promoter in this study. Mutations on our Sryα and Sryγ constructs at amino acid 38 altered promoter regulation for Sryγ only. This suggests that changes to amino acid 38 may compensate for the loss of activity seen when also changing amino acid 76. It should be noted as two amino acids vary between these constructs, Sryα P76T and Sryγ Q38H yield the same mutant proteins as do Sryα H38Q and Sryγ T76P. The *Sry1* promoter regulation for these constructs showed no difference when compared to each other, and serve as an internal control to validate that the changes in regulation could only be due to changes at these two amino acids.

Sryη and Sryθ proteins (produced from the *Sry4* and *Sry4A* loci) show amino acid variation in the bridge domain. The *Sry4* genes have not been cloned and analyzed for expression, so Sryα was chosen for mutagenesis. Amino acid 76 (shown to result in significant functional alterations when a Thr and not a Pro) is conserved among Sryη/Sryθ and Sryα, therefore making Sryα a better choice than the Sryγ or Sryβ protein constructs to use for mutagenesis. Analysis of amino acid variations among the multiple loci, suggests that *Sry4* and *Sry4A* are very old loci as they alone (relative to the other *Sry* copies) share amino acid 83 with mouse (Additional file 1: Figure S10). With the variations of Sryη and Sryθ amino acids falling in the bridge domain, they may alter the interaction with proteins known to interact through the bridge domain of Sry, such as the KRAB containing proteins [26], resulting in altered epigenetic gene silencing of Sryη/Sryθ regulated genes [27].

The C-terminal end contains the largest area of variation among the Sry proteins of *Rattus norvegicus*. There are no known structures for regions outside the HMG box of Sry. With the lack of high sequence conservation of the C-terminal end with other species, it is difficult to determine the importance or functionality of this domain. It has been suggested that the C-terminal end can interact with and stabilize DNA binding [28]; however, it is also likely that this domain serves to recruit other proteins to the DNA. Based on the number of charged amino acids on the surface of the C-terminus (Figure 11A), which also contains a hydrophobic packed structure (Additional file 2), interactions with other proteins (yet to be identified) are possible. The stretch of 13 amino acids deleted in Sryβ alters regulational ability of Sry as does the deletion of the entire Q-rich C-terminal domain. These results support the importance of this region in rat Sry function, even though the region does not share conservation with most other mammalian Sry sequences. It is possible that functions of the Sry N-terminus in all other mammalian species [29,30] have been translocated to the C-terminus of rodent Sry. Since the structure of Sry places both the N and C-terminus flanking the HMG box in the same spatial area, one could easily be functionally substituted for the other.

Current work is underway to compile and annotate the Y-chromosome of the SHR/Akr strain. In labeling the multiple loci of *Sry*, care needs to be taken in naming as we show in this study and others that there are functional variations in the proteins, with differential expression patterns of each. Understanding the mechanisms of the gene duplications seen in this animal model may lend insights into the duplication and transpositions of Sry in many human diseases such as sex reversal. Ultimately, studies such as this allow us to begin to understand evolution of the lesser studied Y-chromosome in an animal model to understand complex diseases such as hypertension, while also addressing the evolution of the Y-chromosome in mammals.

## Conclusions

Currently we know of eleven *Sry* copies on the *Rattus norvegicus* Y-chromosome with additional copies of *Zfy*, *Med14*, *Ccdc110*, and *HMGB1* in this region. Transposable elements seem to play an active role in the duplication and translocation of these genes. The duplication events have led to Sry proteins that have amino acid variations that lead to functional alterations in nuclear localization and transcriptional activity. These results show some of the dynamic processes involved in the evolution of the Y-chromosome and suggest importance in numerous disease models such as hypertension.

## Methods

### Identification of Sry proteins

The sequence of the *Sry1* locus (EU984075) was blasted against the NCBI *Rattus norvegicus* sequences. Protein sequences were translated from BAC clones or sequenced constructs using Expasy translate, and aligned by hand. Several new loci (*Sry3BII*, *Sry4* and *Sry4A*) were confirmed by PCR using primers specific to these loci, JP1R (5’-ccaaatacagcaaggctgag) and JF8L (5’tgtaaggggtaaaagctagtatcc) primer set, with Phusion Hot Start II (Thermo Scientific) on both SHR and WKY genomic DNA (cycling conditions: 98°C-3 min, 98°C -30 sec, 56.8°C -30 sec, 72°C -45 sec, repeats steps 2–4 35 times, 72°C -5 min, 4°C-hold). Reactions were run on 1% agarose gels and the bands were isolated using IBI gel extraction kit (MidSci). Fragments were used in either restriction digest with *Afl* II or a nested PCR using L-BamH1/KozakSry (ctaggatccgaaccatggagggccatgtcaag) and R-Not1-StopcodonSry (ctagcggccgctcgtggaactggtgctgct) with GoTaq polymerase (Promega; cycling conditions: 94°C-4 min, 94°C-1 min,60°C-35 sec,72°C-1 min, repeat steps 2–4 30 times, 72°C-7 min, 4°C-hold).

### Contig design, repeat detection, sequence alignments, and phylogenetics

Contigs were built using the sequenced BACs of Table 1. In short, all sequences were compiled with Sequencher 4.5 (Gene Codes) using max inputs for alignments. For short incomplete BACs, the ends (at least 3000 base pairs) were removed to exclude low sequence depth areas. The previously identified Sry sequences were then aligned to the contig with 100% identity. Repetitive sequences were identified using RepeatMasker [31] with abblast, default speed, rat as the DNA source, no contamination checks, and simple repeats not masked. To compare the rat transposable elements on the Y-chromosome, mouse and human Y-chromosome sequences flanking the respective *Sry* were used (using the DNA source of the respective species for the RepeatMasker analysis). The *Sry* loci were aligned to each of the other loci to identify the conserved fragment size using Clustal Omega [32] with default settings. A minimal conserved fragment size around *Sry* was identified for all the *Sry* loci present in the contigs. Phylogenetics were performed using MEGA-5 [33] on this minimal fragment or the ORF for each of the rat *Sry* loci with *hSRY* [GenBank: CCDS14772] or mSry [GenBank: CCDS30545] using maximum likelihood with the JTT model and 500 bootstrap replications.

### Mutagenesis and promoter cloning

Mutations of the rat Sry pEF-1 expression vectors were created with site directed mutagenesis using Phusion Hot start Taq (Thermo Scientific). The respective primer set for each mutation (F-Sry1H38Q: atcagcaagcagctgggatatcagtgg, R-Sry1H38Q: ctctgaattctgcatgctgggattctg; F-Sry1P76T: aaatatcagactcatcgaagggttaaagtg, R-Sry1P76T: atagtttggatatttctctctgtgtagggt; F-Sry1P83S: tatactttgcagcgtgaagt, R-Sry1P83S: actcctctgtgacactttaa; F-Sry1L98V: ctgcaatgggacaacaacct, R-Sry1L98V: caggttgtacacttttgttgagg; F-Sry3Q38H: atcagcaagcatctgggatatcagtgg, R-Sry3Q38H: ctctgaattctgcatgctgggattctg; F-Sry3T76P: aaatatcagcctcatcgaagggttaaagtg, R-Sry3T76P: atagtttggatatttctctctgtgtagggt; F-hSRYQ93H: atcagcaagcatctgggataccagtgg, R-hSRYQ93H: ctctgagtttcgcattctgggattctc; F-hSRYP131T: aagtatcgaactcgtcggaaggcgaagatg, R-hSRYP131T: ataattcgggtatttctctctgtgcatggc) were phosphorylated with T4 polynucleotide kinase (Fermentas) and used in PCR. T4 DNA ligase (Fermentas) was used to ligate linear vectors and these were transformed into TAM-1 competent *E. coli* (Active Motif). Sryα/γ(del) constructs were created by amplifying Sry from each construct with L-BamH1/KozakSry and R-Not1-StopcodonSry and digested with *Cvi* QI which cuts at base pair 294. The C-terminal end of Sryβ (containing a 13 amino acid deletion from Sryα/γ) was ligated together with the N-terminus of Sryα/γ. For Sryβ(+QR) the N-terminus of Sryβ was ligated with the 13 amino acid larger fragment of Sryα. An HMG only construct (Sryα(HMGbox)) was created by amplifying Sryα with L-BamH1/KozakSry and R-SryXbaBoxOnly (ctctagactgtggcactttaaccc), followed by T4 ligation to re-circularize the vector. Constructs without the C-terminal Q-rich region were created by PCR with L-BamH1/KozakSry and R-SryXba-QR (ctctagatgggtatccagtgg) generating a 142 amino acid protein from each Sry (Sryα(−QR), Sryβ(−QR), Sryγ(−QR)).

The two promoter constructs used in luciferase assays were the Sry1 and AR600 constructs. A pGL3 rat *Sry1* promoter construct (−3317/+10, +1 designating the proposed transcriptional start site) was created by amplification from cloned *Sry1* DNA [GenBank: KC215142] with L-Sry1NheI (5’-CTATGCTAGCTCCATACCAAGAAGGCAGTTG-3’) and R-Sry1HindIII (5’-CGCAAGCTTAAACCCCTGTGGATTGTAAATG-3’). The rat *Sry1* promoter contains multiple potential Sry binding sites as determined by MatInspector (Genomatix). This fragment was cloned into pGL3 with *Nhe* I and *Hind* III restriction digest. The pGL3 AR600 construct (containing the 5’ UTR of AR, with several potential Sry binding sites) was created by PCR with R-ARNco (gtaccatggtttagcttgtctctagcttccacc) and LARSma600 (cacccgggtaactccctttggctga) and cloned in with *Nco* I and *Sma* I restriction digest and ligation. All clones and mutations were sequence confirmed using BigDye Terminator chemistry on ABI 3130xl genetic analyzer (Applied Biosystems).

### Electrophoretic mobility shift assays (EMSA)

Sry1 DNA was inserted into the pIVEX 2.4 with GoTaq (Promega) PCR using 5’- gcgcccgggctagtggaactggtgct and 5’- gcggcccatggagggccatgtcaag with *Nco*I and *Sma* I digests, followed by T4 ligation. Vector was transformed into BL21 (DE3) competent *E. coli* (NEB). A single isolated colony of cells was inoculated into 5 mL terrific broth containing 50 ug/mL Ampicillin and grown to OD_600_ of 0.8 at 37°C. Cells were induced with 0.75 mM IPTG and incubated at 37°C for three hours. Cells were collected at 5000×g for 10 min and frozen at −80°C until lysis. Cells were lysed with 1 mL of 1× CelLytic B (Sigma-Aldrich), 2 mg/mL Lysozyme, 0.25 mM Leupeptin, 1 mM PMSF, 50 Units DNase, 25 mM NaF, 25 mM MgCl in phosphate buffered imidazole. Cells were passed through a 25 gauge needle multiple times to complete lysis and the lysate was centrifuged at 10,000 × g for 20 min.

Freshly annealed primers (5’-CATACTGCGGGGGTGATTGTTCAGGATCATACTGCG-3’ and antisense), containing a 5’ conjugated biotin (IDT) on the sense strand (primers were previously used by another lab to confirm hSRY-DNA binding [34]) were prepared in primer annealing buffer (10 mM Tris, 1 mM EDTA, 100 mM NaCl, pH 8.0) at a final concentration of 10 μM. Annealed primers were diluted to 250 fmol/μL. Non-denaturing TBE polyacrylamide gels (6%) were casted in a 0.75 mm spaced glass cassette (Bio-Rad) with a 10 well comb and pre-electrophoresed for 1 hr at 100 V in 0.5× TBE. Control binding experiments were setup using the LightShift EMSA optimization and control kit (Thermo). All reactions contained 2 μL 10× binding buffer, 1 μL 50% glycerol, 1 μL Poly(dI-dC), and 1 μL 1% NP-40. For the respective reactions, 2 μL Biotin-EBNA control DNA, 2 μL Unlabeled EBNA control DNA, 1 μL EBNA overexpression extract, 2 μL Biotin-Sry-DNA, 2 μL Unlabeled-Sry-DNA (2 pM), and 2 μL Sryα cell lysate sample sizes were used. All reactions were brought to 20 μL total with molecular water. Sryα lysates ranging from 0.5 to 3 μL in 0.5 μL increments were used to show a concentration dependence of DNA binding. Reactions were incubated at room temperature for 20 min. Samples were run on a TBE gel at 100 V for 45 min. A Biodyne B Nylon Membrane (Thermo) was soaked in 0.5× TBE for 10 min. When electrophoresis was complete, the gel was transferred to the membrane in a semi-dry horizontal transfer system at 75 mA for 30 min. The membrane was then dried for twenty minutes at room temperature, cross linked using UV light and detection was performed as recommended in the LightShift Chemiluminescent EMSA kit (Thermo). ImageJ (NIH) was used for densitometry analysis.

### Nuclear localization

CHO-K1 cells (1.25 × 10^4^ cells/cm^2^) used for immunocytology were seeded onto LabTek™ 16 well glass chamber slides (Nunc™) in HAM’s F12K medium (Sigma) supplemented with 10 mM HEPES, 30 mM sodium bicarbonate and 10% fetal bovine serum (Atlanta Biologicals) in a humidified atmosphere at 37°C with 5% CO_2_. After 24 hours cells were transfected with 300 ng plasmid DNA (Sry pEF1expression vector) using 1 μl ExGen500 in 20 μl 150 mM NaCl. After 24 hours of incubation all chambers were washes twice with 1× PBS and fixed for 5 min in ice cold methanol. Cell were incubated 25 min in blocking solution (1× PBS containing 10% normal rabbit serum and 3% nonfat dry milk) followed by incubating one hour with the primary antibodies, goat anti-Myc epitope (Bethyl Laboratories, Inc.) or goat anti-mouse Sry E-19 (Santa Cruz Biotechnology, Inc.) diluted 1:450 and 1:100 respectively in diluted blocking solution (2% normal rabbit serum, 0.6% nonfat milk) at 22°C. After four washes with 1× PBS, the secondary antibody, a rabbit anti-goat IgG-Cy3™ conjugate (Sigma-Aldrich, Inc.) diluted 1:3500 in blocker diluted to 4% normal rabbit serum, was added in 1.2% nonfat milk and incubated 45 min at 37°C. After two washes in 1× PBS, VectaShield mounting medium containing DAPI (Vector Laboratories) was applied and images were captured using a broad range excitation filter (530 – 550 nm) on an Olympus BX60 with DP71 digital camera and DP Controller software. Controls to ensure antibody specificity included CHO transfected as described above with Sryα incubated with normal goat serum or PBS in place of a primary antibody. In all experiments at least 12–25 stained cells were observed per transfection.

CHO-K1 cells were grown to 1.0 × 10^5^ cells/cm^2^ on 100 mm plates (Nunc™) as described previously. Each plate was transfected with 7.5 μg respective Sry pEF1 expression vector that was complexed with 25 μl ExGen500 and 150 mM NaCl was used to bring the total reaction volume to 1 mL. Complexes were applied to CHO in 9 mL fresh medium and centrifuged at 280 × g 5 min. Following a 24 hour incubation, medium was removed, plates were washed with 1× PBS and cells were trypsinized, pelleted (280 × g, 5 min.) and cytoplasmic and nuclear proteins were separated using the reagents and protocol supplied in the ProteoJET™ Cytoplasmic and Nuclear Protein Extraction Kit (Fermentas). Fractions generated were quantified by Bradford Assay (Thermo) and then subjected to SDS PAGE and western blot analysis.

Cytoplasmic and nuclear extracts (20 μg) were separated on 13.5% polyacrylamide gels and proteins were semidry transferred (1 mAmp/cm^2^) to PVDF membranes. Blots were blocked 1 hr at room temperature in 1× TBS containing 5% nonfat dry milk and 0.1% Tween-20. Primary antibodies used to detect Sry proteins include a goat anti-mouse Sry E-19 (Santa Cruz Biotechnology, Inc. ) and a goat anti-Myc epitope (Bethyl Laboratories, Inc. Laboratories). Both antibodies were diluted in blocking solution at 1:300 and 1:1000 respectively and incubated for at least 1 hr at room temperature. Following two washes in 1× PBS, the secondary antibody, donkey anti-goat HRP conjugate (Bethyl Laboratories, Inc. Laboratories) was diluted 1:6,000 in blocking solution and incubated 1 hr at room temperature. Bands were detected using SuperSignal West Pico Chemiluminescent Substrate (Thermo Fisher Scientific Inc.).

### Cotransfections and luciferase assays

Transfection into CHO-K1 cells was performed using 500 ng pGL3 reporter vector, 50 ng of pEF1 expression vector, 500 pg Renilla control vector and 2uL Turbofect (Fermentas) into 5x10^4^ cells preplated in a 24 well plate 24 hours prior to transfections. Cells were lysed 24 hours post transfection and luciferase assays performed with Dual-Luciferase reported assay system (Promega) according to the manufacturer’s instructions. Statistics were performed using JMP software with statistical significance of p < 0.05. ANOVAs were performed followed by individual student’s t-tests. All error bars are presented as the standard error of the mean.

### Modeling protein structures and molecular dynamic simulations

Models of rat Sry tertiary structure were created using I-TASSER [35] and aligned to DNA using the PDB structure 1j46 of human SRY [36] and energy minimized with YASARA [37] with water at 0.998 g/mL and AMBER03 force field [38]. Models for the C-terminal end of Sryα were created with QUARK [39]. The ten models were run in molecular dynamic simulations for 2 nanoseconds and observed for energy and residue movement. The best model (model 4) as determined by maximal hydrophobic packing in md simulation, model quality (determined with the knowledge-based potential of YASARA2), and energy minimization was manually added onto the model of the HMG box bound to DNA, forming the peptide bond, followed by energy minimizations using the AMBER03 force field in water.

### Availability of supporting data

"The data sets supporting the results of this article are available in the GenBank repository, [Accession codes: KC215139, KC215140, KC215141, KC215142; http://www.ncbi.nlm.nih.gov/genbank/]".

## Competing interests

The authors declare that they have no competing interests.

## Authors’ contributions

JWP performed Y-chromosome sequence analysis, newly identified copy number variation detection and sequencing, EMSA and wrote the manuscript. ACU performed nuclear localization studies. JWP and ACU both performed luciferase assays. NM and DP helped in the identification of the new Sry copies, SS in the EMSA assays, and CS in characterization of the Sry1 promoter sequence. MET and AM advised on all studies and contributed significantly to the development of research ideas / conclusions of data. All authors have read and approved the manuscript.

## Supplementary Material

Additional file 1 Supporting data and sequence alignments for the conclusions of the paper. Click here for file

Additional file 2 Model of the HMG box of Sryα bound to DNA with the additional C-terminal domain of Sryα. Click here for file
