# Supplementary material for: Analysis of Sry duplications on the Rattus norvegicus Y-chromosome
Source: BMC Genomics. 2013 Nov 14;14:792. doi: 10.1186/1471-2164-14-792 (PMC3840628; doi:10.1186/1471-2164-14-792)
Supplement: Additional file 1 — Supporting data and sequence alignments for the conclusions of the paper. [file 1471-2164-14-792-S1.pdf]

Supplemental data

```
total length:     432875 bp  (432875 bp excl N/X-runs)
GC level:         41.34 %
bases masked:     235587 bp ( 54.42 %)
==================================================
               number of      length   percentage
               elements*    occupied  of sequence
--------------------------------------------------
SINEs:               144        18304 bp    4.23 %
      Alu/B1          34         3449 bp    0.80 %
      B2-B4           70        11450 bp    2.65 %
      IDs             35         2972 bp    0.69 %
      MIRs             4          376 bp    0.09 %

LINEs:               130       135855 bp   31.38 %
      LINE1          123       134605 bp   31.10 %
      LINE2            6         1208 bp    0.28 %
      L3/CR1           1           42 bp    0.01 %

LTR elements:        144        70554 bp   16.30 %
      ERVL            10         2753 bp    0.64 %
      ERVL-MaLRs      28         9535 bp    2.20 %
      ERV_classI      27        21232 bp    4.90 %
      ERV_classII     79        37034 bp    8.56 %

DNA elements:         11         1812bp     0.42 %
      hAT-Charlie      6          907 bp    0.21 %
      TcMar-Tigger     2          437 bp    0.10 %

Unclassified:         13         6642 bp    1.53 %

Total interspersed repeats:    233167 bp   53.86 %


Small RNA:            14         1250 bp    0.29 %

Satellites:            5         1226 bp    0.28 %
Simple repeats:        0            0 bp    0.00 %
Low complexity:        0            0 bp    0.00 %
==================================================
```

Figure S1 Repeat analysis of Contig 1. Contig 1 of SHR Y-chromosome *Sry* region with repeat analysis done with http://www.repeatmasker.org/

```
total length:     377198 bp  (377198 bp excl N/X-runs)
GC level:         41.85 %
bases masked:     197235 bp ( 52.29 %)
==================================================
               number of      length   percentage
               elements*    occupied  of sequence
--------------------------------------------------
SINEs:                84        9619 bp    2.55 %
      Alu/B1          14        1289 bp    0.34 %
      B2-B4           31        4730 bp    1.25 %
      IDs             38        3567 bp    0.95 %
      MIRs             1          33 bp    0.01 %

LINEs:               100      123589 bp   32.77 %
      LINE1           99      123495 bp   32.74 %
      LINE2            1          94 bp    0.02 %
      L3/CR1           0           0 bp    0.00 %

LTR elements:        120       61699 bp   16.36 %
      ERVL            12        2597 bp    0.69 %
      ERVL-MaLRs       9        3787 bp    1.00 %
      ERV_classI      31       21450 bp    5.69 %
      ERV_classII     68       33865 bp    8.98 %

DNA elements:          1         145bp    0.04 %
      hAT-Charlie      1         145 bp    0.04 %
      TcMar-Tigger     0           0 bp    0.00 %

Unclassified:          1        1029 bp    0.27 %

Total interspersed repeats:   196081 bp   51.98 %


Small RNA:            11        1054 bp    0.28 %

Satellites:            1         100 bp    0.03 %
Simple repeats:        0           0 bp    0.00 %
Low complexity:        0           0 bp    0.00 %
==================================================
```

Figure S2 Repeat analysis of Contig 2. Contig 2 of SHR Y-chromosome *Sry* region with repeat analysis done with http://www.repeatmasker.org/

```
total length:      149399 bp  (149399 bp excl N/X-runs)
GC level:          42.91 %
bases masked:       83452 bp ( 55.86 %)
==================================================
               number of      length   percentage
               elements*    occupied  of sequence
--------------------------------------------------
SINEs:              110        29647 bp   19.84 %
      ALUs          106        29203 bp   19.55 %
      MIRs            4          444 bp    0.30 %

LINEs:               35        28212 bp   18.88 %
      LINE1          32        28047 bp   18.77 %
      LINE2           3          165 bp    0.11 %
      L3/CR1          0            0 bp    0.00 %

LTR elements:        47        22627 bp   15.15 %
      ERVL            4         1439 bp    0.96 %
      ERVL-MaLRs     11         3580 bp    2.40 %
      ERV_classI     32        17608 bp   11.79 %
      ERV_classII     0            0 bp    0.00 %

DNA elements:        16         2864 bp    1.92 %
     hAT-Charlie      7          997 bp    0.67 %
     TcMar-Tigger     4         1397 bp    0.94 %

Unclassified:         0            0 bp    0.00 %

Total interspersed repeats:    83350 bp   55.79 %


Small RNA:            1          102 bp    0.07 %

Satellites:           0            0 bp    0.00 %
Simple repeats:       0            0 bp    0.00 %
Low complexity:       0            0 bp    0.00 %
==================================================
```

Figure S3 Repeat analysis of human SRY region. *hSRY* flanking sequence for 74570bp 3’ and 74829bp 5’ of the A of ATG for *hSRY* transcript.

```
sequences:              1
total length:      197889 bp  (197889 bp excl N/X-runs)
GC level:          39.62 %
bases masked:       98586 bp ( 49.82 %)
==================================================
               number of      length   percentage
               elements*    occupied  of sequence
--------------------------------------------------
SINEs:                 41        5242 bp    2.65 %
      Alu/B1           27        3083 bp    1.56 %
      B2-B4            12        2011 bp    1.02 %
      IDs               0           0 bp    0.00 %
      MIRs              2         148 bp    0.07 %

LINEs:                 32       35022 bp   17.70 %
      LINE1            30       34866 bp   17.62 %
      LINE2             0           0 bp    0.00 %
      L3/CR1            2         156 bp    0.08 %

LTR elements:          93       54808 bp   27.70 %
      ERVL              5        2007 bp    1.01 %
      ERVL-MaLRs       17        4678 bp    2.36 %
      ERV_classI       11       17213 bp    8.70 %
      ERV_classII      60       30910 bp   15.62 %

DNA elements:           4         702bp    0.35 %
      hAT-Charlie       2         418 bp    0.21 %
      TcMar-Tigger      2         284 bp    0.14 %

Unclassified:           2        2006 bp    1.01 %

Total interspersed repeats:     97780 bp   49.41 %


Small RNA:              0           0 bp    0.00 %

Satellites:             5         806 bp    0.41 %
Simple repeats:         0           0 bp    0.00 %
Low complexity:         0           0 bp    0.00 %
==================================================
```

Figure S4 Repeat analysis of mouse Sry region. Analysis of mouse *Sry* sequence for 99311bp 3’ and 98578bp 5’ the A of ATG for the *mSry* transcript.

A

	Sry1	*Sry2*	*Sry3A*	*Sry3B*	*Sry3BII*	*Sry3C*	*Sry4*	*Sry4A*
Sry1	-	421	420	11,600	11,600	11,600	11,600	10,538
Sry2	421	-	420	421	420	424	421	421
Sry3A	8,048	420	-	8,048	8,048	8,048	8,048	8,048
Sry3B	11,600	421	420	-	14,949	14,224	14,675	10,538
Sry3BII	11,600	421	420	15,235	-	14,236	14,675	10,538
Sry3C	11,600	421	420	14,138	13,981	-	14,675	10,538
Sry4	11,600	421	420	14,792	14,506	14,224	-	10,538
Sry4A	10,555	421	420	10,570	10,532	10,719	10,694	-

B

	Sry1	*Sry2*	*Sry3A*	*Sry3B*	*Sry3BII*	*Sry3C*	*Sry4*	*Sry4A*
Sry1	-	12,822	1,579	1,974	1,969	12,766	957	989
Sry2	12,763	-	1,579	1,974	1969	30,907	957	989
Sry3A	1,579	1,579	-	1,579	1,579	1,579	957	989
Sry3B	1,975	1936	1,579	-	17,731	1,971	15,252	15,407
Sry3BII	1,975	1936	1,579	15,482	-	1,971	20,389	22,094
Sry3C	12,763	24,564	1,579	1,974	1,969	-	957	957
Sry4	968	924	959	15,482	23,197	961	-	43,175
Sry4A	968	924	959	15,456	23,634	961	42,847	-

C

	Sry1	*Sry2*	*Sry3A*	*Sry3B*	*Sry3BII*	*Sry3C*	*Sry4*	*Sry4A*
Sry1	-	13,243	1,999	13,574	13,569	24,366	12,557	11,527
Sry2	13,184	-	1,999	2,395	2,389	31,331	1,378	1,410
Sry3A	9,627	1,999	-	9,627	9,627	9,627	9,005	9,037
Sry3B	13,575	2,357	1,999	-	32,680	16,195	29,927	25,945
Sry3BII	13,575	2,357	1,999	30,717	-	16,207	35,064	32,632
Sry3C	24,363	24,985	1,999	16,112	15,950	-	15,632	11,495
Sry4	12,568	1,345	1,379	30,274	37,703	15,185	-	53,713
Sry4A	11,523	1,345	1,379	26,026	34,166	11,680	53,541	-

Figure S5 Conservation analysis of the *Sry* copies. A) Base pairs conserved 5’ of the A of ATG. Highlighted in red are constructs involving Sry1 where conservation goes to the end of the contig. Highlighted in cyan are those sequences that have a 6,809 bp transposable element inserted causing a disruption of homology that picks back up for 7,647 bp. Numbers in red are those that define the size fragment for phylogenetics. B) Base pairs conserved 3’ of the A of ATG, with the green highlighted box showing a 6,386 bp insertion disrupting homology. C). Total conserved fragment length 5’ and 3’ of the A of ATG. At the top of each chart is the gene that the sequence was aligned to the respective *Sry* on the side.

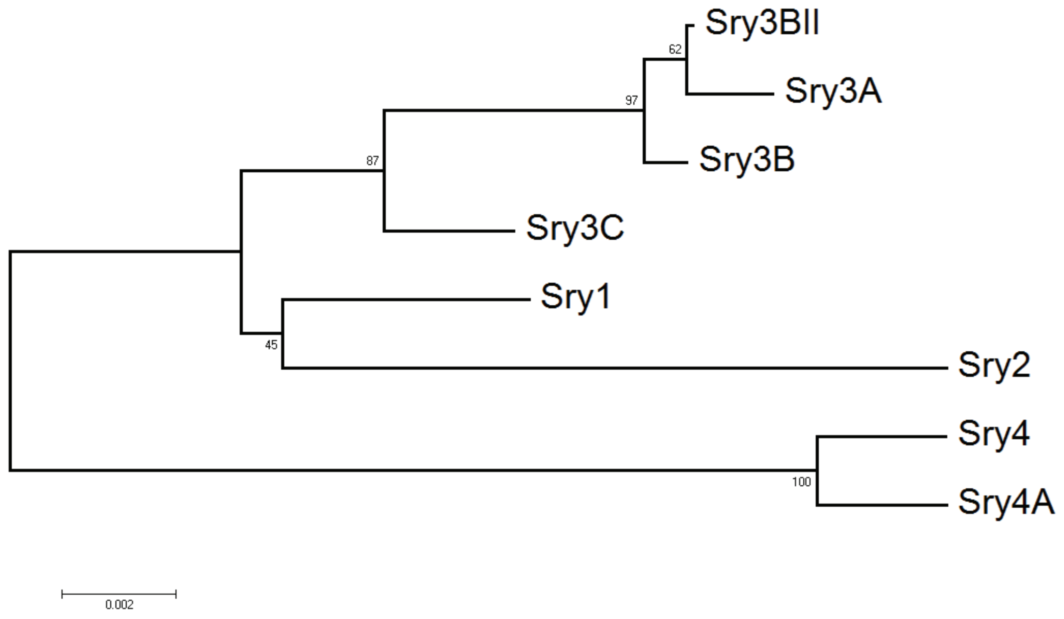

Figure S6 Phylogenetic tree of the maximally conserved fragment of Sry copies. Phylogenetic tree using *Sry* genes of the maximum conserved fragment based on contig analysis of Figure S5. Numbers next to each node represent the percent of the 500 bootstrap phylogenies that were identified similar to the one shown in the figure.

A

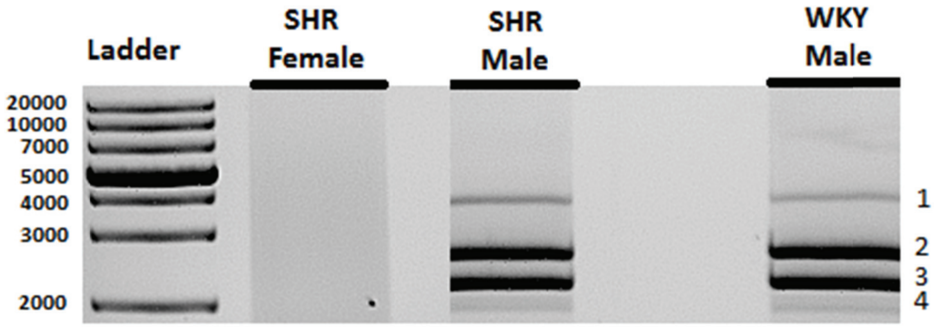

B

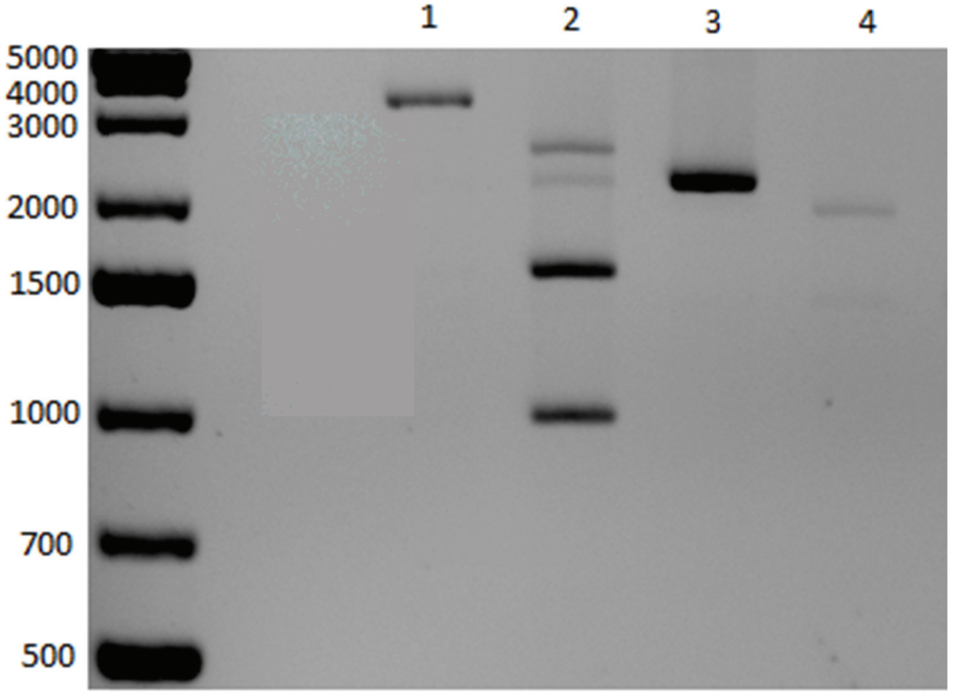

Figure S7 PCR products after amplification with JP1R and JF8L specific primers. **A**) Gel of PCR amplicons from SHR and WKY genomic DNA, showing the presence of Sry3BII (4220bp, band 1), Sry4A (2031 bp, band 3), and Sry4 (2003, band 4). Band 2 is believed to be nonspecific amplification of both Sry4A and 4 based on primer mispriming around 400 base pairs upstream where JF8L should bind. **B**). Digestion of each band with AflII. Bands 1 and 3 when digested with *Afl*II did not digest since there is no *Afl*II site found in the DNA fragments produced when amplifying either Sry3BII or Sry4A. Band 4 partially digested, suggesting further proof that it is Sry4 which contains an *Afl*II site. Band two showed similar digestion pattern as seen in band 4.

```
Med14Y   AGCCAGGCTGTGCGCCGCCATGGCTCCAGTGCAGCTGGACAACCACCAGCTCATCCCTCC
Med14X   -------------------ATGGCCCCAGTGCAGCTGGAGAACCACCAGCTGATCCCTCC
                            ***** ************** *********** ********

Med14Y   TGGCCGCGGAGGTGGCAGAGGTGGCGGCGAAGGCAGCAGCAGGGACTTGGTGTCTGCCCC
Med14X   TGGCGGCGGAGGTGGCAGCGGTGGCGGTGGAGGCAGCAGCAGCGGCTCTGCGTCTGTCCC
         **** *************.******** *.************ *.**  * ***** ***

Med14Y   GGCTCCCCCATCGCCCGGAGCCAT---GGCGGTGGCAGCAGCCTCCGCGACTACCCCTGG
Med14X   GGCTCCCCCGCCTCCTGGAGCCGCTGTGGCGGCGGCAGCTGCGGCTGCTGCTAGCCCTGG
         *********. * ** ******.    ***** ******:**  * ** .*** ******

Med14Y   ATACCGGCTTAGCACACTCATCGAATTTTTGCTGCACAGGGCCTACTCAGAGCTTATGGT
Med14X   ATACCGGCTTAGCACGCTCATCGAATTTCTGCTGCACCGGGCCTACTCCGAGCTTATGGT
         ***************.************ ********.**********.***********

Med14Y   GTTGACGGACTTCTTGCCAAGGAAATCTGATGTGGAAAGGAAAATAGAAATTGTACAGTT
Med14X   GTTGACGGACTTATTGCCAAGAAAATCGGATGTGGAAAGGAAAATAGAAATTGTACAGTT
         ************.********.***** ********************************

Med14Y   TGCTAGCCGGACACGTCAACTCTTCGTTCGGTTATTAGCTTTAGTAAAATGGGCCAGTGA
Med14X   TGCTAGCCGGACACGTCAACTCTTCGTTCGATTATTAGCTTTAGTAAAATGGGCCAATGA
         ******************************.*************************.***

Med14Y   TGCTGGCAAGGTAGAAAAGTGTGCGATGATCACAAGCTTTTTAGATCATCAAGCCATCTT
Med14X   TGCTGGCAAAGTAGAAAAGTGTGCGATGATCTCGAGCTTTTTAGACCAGCAAGCCATCTT
         *********.*********************:*.*********** ** ***********

Med14Y   ATTTGTGGATACCGCTGACCGCTTGGCCTCCTTAGCTAGAGATGCCCTGGTCCATGCCTG
Med14X   ATTTGTGGATACTGCTGACCGCTTGGCCTCCTTAGCTAGAGATGCCCTGGTCCATGCACG
         ************ ********************************************. *

Med14Y   CCTGCCTAGTTTTGCTATCCCATATGCCATTGATGTACTGACTACTGGCTCTTATCCAAG
Med14X   CCTGCCTAGTTTTGCTATCCCATATGCCATTGATGTACTGACTACTGGTTCTTATCCACG
         ************************************************ *********.*

Med14Y   GCTTTCAACCTGCATCAGGGACAAAATTATTCCTCCAGACCCTATTACCAAGATTGAGAA
Med14X   ACTTCCAACCTGCATCAGAGACAAAATTATTCCTCCAGACCCAATTACCAAAATTGAGAA
         .*** *************.***********************:********.********

Med14Y   ACAAGCCGCACTTCATCAGCTTAATCAGATTCTTAGACATAGGCTTGTAACAGCAGATCT
Med14X   ACAAGCCACACTTCATCAGCTTAATCAGATTCTTAGACATAGGCTTGTAACGACAGATCT
         *******.*******************************************..*******

Med14Y   TCCTCCACAGCTAGCAAATCTTACAGTGGCGAATGGCCGTGTGAAGTTTCGAGTTGAAAG
Med14X   TCCTCCACAGCTAGCAAATCTTACAGTGGCAAATGGCCGTGTGAAGTTTCGAGTTGAAGG
         ******************************.***************************.*

Med14Y   AGAATTTGAAGCAACCTTGACAGTGATGGGTGATGACCCAAAAGTCCCATGGTGTCTTCT
Med14X   AGAATTTGAAGCAACCTTGACGGTGATGGGTGATGACCCAGAAGTCCCATGGCGTCTTCT
         *********************.******************.*********** *******
```

Figure S8 Med14Y. Alignment of the DNA coded on the SHR Y-chromosome (Med14Y) to the mRNA of Med14 on the X-chromosome (NCBI Gene ID: 317343). Sequence of the multiple exons are highlighted in rotating yellow and red to allow for their identification.

```
Med14Y   CAAGCTAGAAATTCTAGTTGAGGATAAGCAAACGGGAGATGGGCGAGCTTTGGTTCATAG
Med14X   CAAGCTAGAAATTCTAGTTGAGGATAAGGAAACAGGAGATGGGCGAGCTTTGGTTCATAG
         **************************** ****.**************************

Med14Y   CATGCAAATTGACTTTATCCATCAGCTAGTCCAGTCCAGGCTCTTTGCTGATGAGAAACC
Med14X   CATGCAAATTGACTTTATCCATCAGCTGGTCCAGTCTAGGCTCTTTGCTGATGAGAAACC
         ***************************.******** ***********************

Med14Y   TCTTCAGGACATGTACAACTGCCTGCATTTTTTCTGCTTATCGCTTCAATTAGAAGTATT
Med14X   TCTTCAGGACATGTACAACTGCCTACATTGTTTCTGCTTATCACTTCAATTAGAAGTCTT
         ************************.**** ************.**************.**

Med14Y   ACATTCCCAGACGCTAATGTTAATCAGAGAGAGGTGGGGAGACCTTGTACAGGTGGAAAG
Med14X   ACATTCCCAGACACTAATGTTAATCCGAGAGAGGTGGGGAGACCTTGTACAGGTGGAAAG
         ************.************.**********************************

Med14Y   ATACCGTGCTGGAAAGTGCCTC------TCGGTTTGGAGCCAACAGGTTCTAGGGAAAAA
Med14X   ATACCATGCTGGAAAGTGCCTCTCCCTCTCAGTTTGGAATCAACAGGTTCTAGGGAGAAA
         *****.****************      **.*******. ****************.***

Med14Y   AACAGGCACAGCATCTGTTCACAAAGTTACAATTAAAATCGATGAGAATGATGTCTCCAA
Med14X   AACAGGCACAGCATCTGTTCACAAAGTTACAATTAAAATCGATGAGAATGATGTCTCCAA
         ************************************************************

Med14Y   GCCTTTACAGATTTTCCACGATCCTCCTTTACCAGCTTCTGATTCTAAATTAGTAGAAAG
Med14X   GCCTTTACAGATTTTTCATGATCCTCCTTTGCCAGCTTCTGATTCTAAATTAGTAGAAAG
         *************** ** ***********.*****************************

Med14Y   AGCCATGAAGATTGACCACTTATCAATAGAAAAGCTCCTGATTGACAGTGTACACTCACG
Med14X   AGCCATGAAGATTGACCACTTATCAATAGAAAAGCTCCTGATTGACAGTGTACACGCTAG
         ******************************************************* *:.*

Med14Y   AGCCCACCAGAAGCTGCAGGAACTGAAGGCCATTCTTAGAAGCTTCAATGCTAATGAAAA
Med14X   AGCCCACCAGAAGCTGCAGGAACTGAAGGCCATTCTTAGAAGCTTCAATGCCAACGAAAA
         *************************************************** ** *****

Med14Y   CTCTTCCATAGAGACTGCACTTCCAGCTCTTATTGTACCCATCTTGGAGCCCTGTGGTAA
Med14X   CTCTTCCATAGAGACTGCACTTCCAGCTCTTATTGTGCCCATCTTGGAGCCCTGTGGTAA
         ************************************.***********************

Med14Y   TTCGGAGTGCCTGCACATTTTTGTAGATTTGCATTCTGGAATGTTCCAATTGATGCTTTA
Med14X   TTCGGAGTGCCTACACATTTTTGTAGATTTGCATTCTGGAATGTTCCAATTGATGCTTTA
         ************.***********************************************

Med14Y   TGGACTTGACCAGGCCACTCTGGAGGACATGGAAAAATCGCTGAATGATGACATGAAGCG
Med14X   TGGACTTGATCAGGCCACTCTGGAGGACATGGAAAAGTCTTTGAATGATGACATGAAGCG
         ********* **************************.**  *******************

Med14Y   GATCATCCCTTGGATTCAACAACTTAAGTTTTGGCTGGGACAACAGCGTTGCAAACAATC
Med14X   GATCATCCCTTGGATTCAGCAACTTAAGTTTTGGCTGGGACAACAGCGTTGCAAACAATC
         ******************.*****************************************

Med14Y   TATAAAACATCTGCCTGCAATAACCACTGAAACGTTGCAGCTCGCCAACTATTCAACACA
Med14X   TATAAAACATCTGCCTACCATAACCACTGAAACGTTGCAGCTTGCCAACTATGCAACACA
         ****************.*.*********************** ********* *******
```

Figure S8 Med14Y. Alignment of the DNA coded on the SHR Y-chromosome (Med14Y) to the mRNA of Med14 on the X-chromosome (NCBI Gene ID: 317343). Sequence of the multiple exons are highlighted in rotating yellow and red to allow for their identification.

```
Med14Y   TCCCATTGGTAGGCTTTCTAAGAACAAGTTGTTTATTAAACTTACTCGCCTTCCGCAGTA
Med14X   TCCCATTGGAAGCCTGTCTAAGAATAAGCTGTTTATTAAACTTACTCGCCTTCCACAGTA
         *********:** ** ******** *** *************************.*****

Med14Y   CTACATTGTTGTGGAAATGCTTGAGGTTCCTAATAAGCCCACCCAGCTATCATACAAGTA
Med14X   CTACATTGTTGTGGAAATGCTTGAGGTTCCTAATAAGCCCACACAGCTATCGTACAAGTA
         ******************************************.********.********

Med14Y   TTACTTTATGTCTGTGAGTACTGCAGATGGCGAGAACACACCTGTCATGGCACTGCTGCT
Med14X   TTACTTTATGTCTGTGAGTACTGCAGATCGTGACGACAGCCCTGCCATGGCACTGCTGCT
         **************************** * ** .*** .**** ***************

Med14Y   GCAGCAATTCAAGGACAACATCCAGGACTTGATGTCCTATACAAAGATTGGAAAACAGAC
Med14X   GCAGCAATTCAAGGACAACATCCAGGACTTGATGTCCTGTACAAACACTGGGAAACAGAC
         **************************************.****** * ***.********

Med14Y   CATAATTGGTACCAAGCACAACTTATCTGATGACCCATGTCCTGTAGAATGCAAGAAAGC
Med14X   AAGAACTGGTACCAAGCACAAGTTGTCTGATGACTCGTGCCCGGTAGAATGTAAGAAAGC
         .* ** *************** **.********* *.** ** ******** ********

Med14Y   CAAACAATCAGGAGAAATGTGTGCCTTCAATAAAGTTCTAGCTCACTTTGTCGCTATGTG
Med14X   CAAACGATCAGGAGAAACGTGTGCCTTCAATAAAGTTCTAGCTCACTTTGTCGCTATGTG
         *****.*********** ******************************************

Med14Y   TGACACCAACATGCCATTTGTAGGACTTCGATTGGAGTTGTCCAACCTGGAGATACCACA
Med14X   TGATACCAACATGCCATTTGTAGGACTTCGATTGGAGTTGTCCAATTTGGAGATACCACA
         *** *****************************************  *************

Med14Y   TCAGGGAGTACAAGTGGAAGGTGACGGCTTCAGCTATGCAATTCGCTTACTAAAAATTCC
Med14X   TCAGGGAGTGCAAGTGGAAGGTGATGGCTTCAGCCATGCAATTCGCTTATTAAAAATTCC
         *********.************** ********* ************** **********

Med14Y   TCCCTGTAAGGGAATAAGTGAGGAAACGCAGAAGGCTCTGGACTGCTCTCTTCTTGATTG
Med14X   TCCCTGTAAAGGAATCAGTGAGGAAACACAAAAGGCCCTGGACCGTTCTCTTCTTGATTG
         *********.*****.***********.**.***** ****** * **************

Med14Y   CACTTTCCGATTACAAGGTAGAAATAACCGCACATGGGTGGCAGAGTTAGTGTTTGCAAA
Med14X   CACTTTCCGATTACAAGGTAGAAATAACCGCACATGGGTGGCAGAGTTAGTGTTTGCAAA
         ************************************************************

Med14Y   TTGTCCTCTTAATGGCACTTCTATCAGGGAGCAAGGACCATCCAGACATGTTTACCTGAC
Med14X   TTGTCCGCTTAATGGCACTTCTACCAGGGAGCAAGGACCATCCCGACATGTTTACCTGAC
         ****** **************** *******************.****************

Med14Y   ATATGAAAATCTGTTGTCTGAACCTGTTGGTGGTAGAAGAGTAGTTGAAATGTTTCTTAA
Med14X   ATATGAAAATCTGTTGTCTGAACCTGTTGGCGGTAGAAAAGTAGTTGAAATGTTTCTTAA
         ****************************** *******.*********************

Med14Y   TGACTGGAGTAGCATTTCCCGATTATATGAGTGTGTGTTGGAATTTGCACATTCTCTACC
Med14X   TGACTGGAGTAGCATTGCCCGATTATATGAGTGTGTGTTGGAATTTGCACGTTCTCTACC
         **************** *********************************.*********

Med14Y   AGAAATACCTGCTCATCTGAATTTTTTCTCAGAAGTTTGTGTTTACAATTATCGAAAACT
Med14X   AGAAATACCTGCTCATCTGAATATTTTCTCAGAAGTTCGTGTTTATAATTATCGCAAACT
         **********************:************** ******* ********.*****
```

Figure S8 Med14Y. Alignment of the DNA coded on the SHR Y-chromosome (Med14Y) to the mRNA of Med14 on the X-chromosome (NCBI Gene ID: 317343). Sequence of the multiple exons are highlighted in rotating yellow and red to allow for their identification.

```
Med14Y    TATCTTGTGTTATGGAACCACCAGAGGAAGCTCAATCAGTATCCAGTGGAATTCCATTCA
Med14X    TATCTTGTGTTATGGAACCACCAAAGGAAGTTCAATTAGTATCCAGTGGAATTCCATTCA
          **********************.******* ***** ***********************

Med14Y    TCAGAAATTTTACATTTCTTTGGGAACAGTTGGCCCAAACTCAGGTTGCAGTAATTGTCA
Med14X    TCAGAAATTTCACATTTCTTTGGGAACAGTTGGCCCAAACTCAGGTTGCAGTAACTGTCA
          ********** ******************************************* *****

Med14Y    TATTACCATTCTCCATCACCTTCAAGAAATGTTCAACAAAACACCAAATGTGGTCCAGTT
Med14X    CAATACCATTCTCCATCAGCTTCAAGAAATGTTCAACAAAACACCAAATGTGGTTCAGTT
           *:*************** *********************************** *****

Med14Y    ATTACAGGTTTTGTTTGATACTCAGGCACCATTAAATGCCATCAACAAACTCCCTACTGT
Med14X    ATTACAGGTTTTGTTCGATACTCAAGCACCATTAAATGCCATCAACAAACTCCCTACTGT
          *************** ********.***********************************

Med14Y    TCCAGTGCTGGGCTTGACTCAGAGAAGTAACACCGCCTACCAGTGTTTCTCCATATTACC
Med14X    CCCAATGTTGGGCTTGACTCAGAGAACTAACACCGCCTACCAGTGTTTCTCCATATTACC
           ***.** ****************** *********************************

Med14Y    ACATTCATCCACCCACATCAGATTGGCCTTCAGGAACATGTACTGCATTGATATATACTG
Med14X    ACAATCATCCACCCACATCAGACTGGCCTTCAGGAACATGTACTGCATTGACATATACTG
          ***:****************** **************************** ********

Med14Y    CCTTAGTCACGGTGTCGTGGCAGTACGGGACGGTGCCTATAGTCTTTGTGATAACAGCAA
Med14X    CCGTAGTCGAGGTGTCGTGGCGATACGGGACGGTGCCTATAGTCTTTTTGATAACAGCAA
          ** *****..***********..************************ ************

Med14Y    GTTAGTTGAAGGCTTCTGTCCTGCACCAGGATTAAAGACTTTCCTGAACATGTTTGTTG-
Med14X    GTTAGTTGAAGGCTTCTATCCTGCACCAGGATTAAAGACTTTCCTGAACATGTTTGTGGA
          *****************.*************************************** * 

Med14Y    -----------ATGCTCAGAAAAGAGCTTTAAAGGAGGATGATTACCCTCCTTCTCCTAT
Med14X    TAGCAATCAAGATGCTCGGAGAAGATCTGTCAATGAGGATGATAACCCTCCTTCTCCTAT
                     ******.**.**** ** *.** *********:****************

Med14Y    AGGAGGAGATAGGACGGACTCTTTAATATCACGACTCCAGCCACCTCAGCAACAGCCATT
Med14X    AGGAGGTGACATGATGGACTCTTTAATATCACAGCTCCAGCCACCTCAGCAACAGCCATT
          ******:** * ** *****************..**************************

Med14Y    TCCAAAGCAGCCAGGGATATCGGGCGCTTACCCTCTTACTTCATCTCCTACATCCTATCG
Med14X    TCCAAAGCAGCCAGGGACATCAGGCGCTTACCCTCTTACTTCACCCCCTGCATCCTATCA
          ***************** ***.********************* * ***.*********.

Med14Y    CAGCCCAGTTAATCAGCCCCCCTCTATGATGCACACACAGCCTCCAG-------------
Med14X    CAGCACAGTTAATCAGTCCCCCTCTATGATGCACACACAGTCTCCAGGAAATCTGCATGC
          ****.*********** *********************** ******

Med14Y    ------------------------------------------------------------
Med14X    TGCCAGCTCCCCCAGTGGGGCTTTGAGAGCCCCATCACCAGCGTCATTTGTTCCAACTCC

Med14Y    -----------------------------------------------------------G
Med14X    TCCCCCGTCCTCGCATGGAATCTCAATAGGACCAGGGGCCAGTTTGCTAGTCCACATGG
                                                                     *
```

Figure S8 Med14Y. Alignment of the DNA coded on the SHR Y-chromosome (Med14Y) to the mRNA of Med14 on the X-chromosome (NCBI Gene ID: 317343). Sequence of the multiple exons are highlighted in rotating yellow and red to allow for their identification.

```
Med14Y   AGCCCTTGACTCTAGTTCTCCATACACTCTTATGTCATCAAGTGGA--------------
Med14X   AGCCCTTGACCCTAGTTCTCCATACACTATGGTGTCACCAAGTGGACGAGCAGGAAACTG
         ********** *****************.* .***** ********

Med14Y   ----GGGTCTCCTCCAGTGTTTGTGTCCAGACTCTCACCAGCAACCCCTTTGCCTGGAAT
Med14X   GCCTGGGTCTCCTCAA------GTGTCGGGACCCTCACCAGCAACCCGTTTGCCCGGAAT
             **********.*      ***** .*** ************** ****** *****

Med14Y   GTCACCAGCTAACCCATCTCTGCATTCAACTGTCCCAGATGTTTTTCATTCCCCTCGAGC
Med14X   GTCACCAGCTAACCCATCTCTGCATTCTCCTGTTCCAGATGTTTCTCATTCCCCTCGAGC
         ***************************:.**** ********** ***************

Med14Y   TGGCACAAGTTCCCAGACAATGCCAACCAACATGTCTCCACCTCGAAAACTACCTCAGCG
Med14X   AGGCACAAGTTCCCAGACAATGCCAACCAACATGCCTCCACCTCGAAAACTACCTCAGCG
         :********************************** *************************

Med14Y   CTCCTGTGCAGCATCTGTACCCACCATTCTCACTCACAGTGCCTTGAACATCTTACTGCT
Med14X   CTCCTGGGCAGCATCCATACCCACCATTCTCACTCATAGTGCCTTGAACATCTTACTGCT
         ****** ******** .******************* ***********************

Med14Y   ACCTTCTCCAATGCCAGGCCTTGTGCCTGGCCTGGCAGGTAGTTACCTTTGTTCCCCACT
Med14X   ACCCTCTCCAACGCCAGGCCTTGTGCCTGGCCTGGCAGGTAGTTACCTTTGTTCCCCACT
         *** ******* ************************************************

Med14Y   TGAGAGATTCCTTGGGTCTGTCATCATGAGACGACACCTTCAAAGAATTATCCAACAGGA
Med14X   CGAGAGATTCCTTGGCTCTGTCATCATGAGACGACACCTTCAAAGAATTATCCAACAGGA
          ************** ********************************************

Med14Y   GACATTGCAGCTGATCAGTTCCAATGAACCTGGTGTAATCATGTTCAAGACAGATGCCCT
Med14X   AACGTTGCAGCTGATCAATTCCAATGAACCTGGTGTAATCATGTTCAAGACAGATGCCCT
         .**.*************.******************************************

Med14Y   GAAATGCAGAGTAACCCTTAGTCCCCAAACCAACCAGACCCTTCAGCTAAAAGTGACACC
Med14X   GAAGTGCAGAGTAGCCCTTAGTCCCAAAACCAACCAGACCCTTCAGCTAAAAGTGACACC
         ***.*********.***********.**********************************

Med14Y   TGAAAATGTAGGACAGTGGAAACCTGATGAGCTTCAAGTTTTGGAGAAATTCTTTGAAAC
Med14X   TGAAAATGCAGGACAATGGAAACCTGATGAGCTTCAAGTTTTGGAGAAATTCTTCGAAAC
         ******** ******.************************************** *****

Med14Y   AAGAGTTGCAGGACCACCATTTAAAGCCAACACACTTATAGCCTTCACCAAGCTGTTGGG
Med14X   AAGAGTTGCAGGACCACCGTTTAAAGCCAACACACTTATAGCCTTCACCAAGCTGTTGGG
         ******************.*****************************************

Med14Y   AGCGCCAACACACATCCTCCGGGACTGTGTGCATATAATGCAGTTGGAGGTGTTCCCAGA
Med14X   AGCACCGACACACATTCTCCGGGACTGTGTGCATATCATGAAGTTGGAGCTGTTCCCAGA
         ***.**.******** ********************.***.******** **********

Med14Y   CCAAGCAACCCAGCTGAAATGGAACGTTCAATTTTGCCTCACCATCCCTCCTAGTGCACC
Med14X   CCAAGCAACGCAGCTAAAGTGGAATGTTCAATTTTGCCTCACCATCCCTCCCAGCGCGCC
         ********* *****.**.***** ************************** ** **.**

Med14Y   ACCAATTGCAGCTCCTGGGACACCAGCCGTGGTCCTGAAATCCAAAATGCTGTTTTTTCT
Med14X   ACCCATTGCACCTCCTGGGACACCAGCTGTGGTCCTGAAATCCAAAATGCTCTTTTTTCT
         ***.****** **************** *********************** ********
```

Figure S8 Med14Y. Alignment of the DNA coded on the SHR Y-chromosome (Med14Y) to the mRNA of Med14 on the X-chromosome (NCBI Gene ID: 317343). Sequence of the multiple exons are highlighted in rotating yellow and red to allow for their identification.

```
Med14Y   TCAGCTAACCCAGAAAACATCAGTCCCTCCCCAAGAACCTGTTAGTATAATAGTTCCAAT
Med14X   TCAACTAACTCAGAAAACATCAGTCCCTCCCCAAGAACCTGTTAGTATAATAGTTCCAAT
         ***.***** **************************************************

Med14Y   TATTTATGATATGGCTTCAGGTATCACCCAGCAGGCAGACATTCCCAGACAGCAGAACTC
Med14X   TATTTATGATATGGCTTCAGGTACCACTCAGCAGGCAGACATTCCCAGACAGCAGAACTC
         *********************** *** ********************************

Med14Y   TTCTGTTGCTGCCCCAATGATGGTCAGCACCATTCTGAAGAGGTTTGCAGAGATGAACCC
Med14X   TTCTGTTGCTGCGCCAATGATGGTCAGCAACATTCTGAAGAGGTTTGCAGAGATGAACCC
         ************ ****************.******************************

Med14Y   TCCACGGCAAGGTGAATGTACAATATTTGCAGCTGTTTGTGATTTAATGGCACATCTTAC
Med14X   TCCACGACAAGGTGAATGCACAATATTTGCAGCTGTTCGTGATTTAATGGCTAATCTTAC
         ******.*********** ****************** *************:.*******

Med14Y   ACTGCCCCCTGGTGGGTGTCCATAGACCCAATTGATTTTAAACCAGGAAGGCTGACAAAT
Med14X   ACTGCCCCCTGGTGGGCGTCCATAGACACTATTGTTTTTAAACCAGGAAGGCTGACAAAT
         **************** **********.*:****:*************************
```

Figure S8 Med14Y. Alignment of the DNA coded on the SHR Y-chromosome (Med14Y) to the mRNA of Med14 on the X-chromosome (NCBI Gene ID: 317343). Sequence of the multiple exons are highlighted in rotating yellow and red to allow for their identification.

```
Med14Y                    MAPVQLDNHQLIPPGRGGGRGGGEGSSRDLVSAPAPPSPG-AMAVAAASATTPGYRLSTL 59
Med14X                    MAPVQLENHQLIPPGGGGGSGGGGGSSSGSASVPAPPPPGAAVAAAAAAAASPGYRLSTL 60
                          ******:******** *** *** *** . .*.****.** *:*.***:*::********

Med14Y                    IEFLLHRAYSELMVLTDFLPRKSDVERKIEIVQFASRTRQLFVRLLALVKWASDAGKVEK 119
Med14X                    IEFLLHRAYSELMVLTDLLPRKSDVERKIEIVQFASRTRQLFVRLLALVKWANDAGKVEK 120
                          *****************:**********************************.*******

Med14Y                    CAMITSFLDHQAILFVDTADRLASLARDALVHACLPSFAIPYAIDVLTTGSYPRLSTCIR 179
Med14X                    CAMISSFLDQQAILFVDTADRLASLARDALVHARLPSFAIPYAIDVLTTGSYPRLPTCIR 180
                          ****:****:*********************** *********************.****

Med14Y                    DKIIPPDPITKIEKQAALHQLNQILRHRLVTADLPPQLANLTVANGRVKFRVEREFEATL 239
Med14X                    DKIIPPDPITKIEKQATLHQLNQILRHRLVTTDLPPQLANLTVANGRVKFRVEGEFEATL 240
                          ****************:**************:********************* ******

Med14Y                    TVMGDDPKVPWCLLKLEILVEDKQTGDGRALVHSMQIDFIHQLVQSRLFADEKPLQDMYN 299
Med14X                    TVMGDDPEVPWRLLKLEILVEDKETGDGRALVHSMQIDFIHQLVQSRLFADEKPLQDMYN 300
                          *******:*** ***********:************************************

Med14Y                    CLHFFCLSLQLEVLHSQTLMLIRERWGDLVQVERYRAGKCLS--VWSQQVLGKKTGTASV 357
Med14X                    CLHCFCLSLQLEVLHSQTLMLIRERWGDLVQVERYHAGKCLSLSVWNQQVLGRKTGTASV 360
                          *** *******************************:******  **.*****:*******

Med14Y                    HKVTIKIDENDVSKPLQIFHDPPLPASDSKLVERAMKIDHLSIEKLLIDSVHSRAHQKLQ 417
Med14X                    HKVTIKIDENDVSKPLQIFHDPPLPASDSKLVERAMKIDHLSIEKLLIDSVHARAHQKLQ 420
                          ****************************************************:*******

Med14Y                    ELKAILRSFNANENSSIETALPALIVPILEPCGNSECLHIFVDLHSGMFQLMLYGLDQAT 477
Med14X                    ELKAILRSFNANENSSIETALPALIVPILEPCGNSECLHIFVDLHSGMFQLMLYGLDQAT 480
                          ************************************************************

Med14Y                    LEDMEKSLNDDMKRIIPWIQQLKFWLGQQRCKQSIKHLPAITTETLQLANYSTHPIGRLS 537
Med14X                    LEDMEKSLNDDMKRIIPWIQQLKFWLGQQRCKQSIKHLPTITTETLQLANYATHPIGSLS 540
                          ***************************************:***********:***** **

Med14Y                    KNKLFIKLTRLPQYYIVVEMLEVPNKPTQLSYKYYFMSVSTADGENTPVMALLLQQFKDN 597
Med14X                    KNKLFIKLTRLPQYYIVVEMLEVPNKPTQLSYKYYFMSVSTADRDDSPAMALLLQQFKDN 600
                          ******************************************* :::*.***********

Med14Y                    IQDLMSYTKIGKQTIIGTKHNLSDDPCPVECKKAKQSGEMCAFNKVLAHFVAMCDTNMPF 657
Med14X                    IQDLMSCTNTGKQTRTGTKHKLSDDSCPVECKKAKRSGETCAFNKVLAHFVAMCDTNMPF 660
                          ****** *: ****  ****:****.*********:*** ********************

Med14Y                    VGLRLELSNLEIPHQGVQVEGDGFSYAIRLLKIPPCKGISEETQKALDCSLLDCTFRLQG 717
Med14X                    VGLRLELSNLEIPHQGVQVEGDGFSHAIRLLKIPPCKGISEETQKALDRSLLDCTFRLQG 720
                          *************************:********************** ***********

Med14Y                    RNNRTWVAELVFANCPLNGTSIREQGPSRHVYLTYENLLSEPVGGRRVVEMFLNDWSSIS 777
Med14X                    RNNRTWVAELVFANCPLNGTSTREQGPSRHVYLTYENLLSEPVGGRKVVEMFLNDWSSIA 780
                          ********************* ************************:************:

Med14Y                    RLYECVLEFAHSLPEIPAHLNFFSEVCVYNYRKLILCYGTTRGSSISIQWNSIHQKFYIS 837
Med14X                    RLYECVLEFARSLPEIPAHLNIFSEVRVYNYRKLILCYGTTKGSSISIQWNSIHQKFHIS 840
                          **********:**********:**** **************:***************:**

Med14Y                    LGTVGPNSGCSNCHITILHHLQEMFNKTPNVVQLLQVLFDTQAPLNAINKLPTVPVLGLT 897
Med14X                    LGTVGPNSGCSNCHNTILHQLQEMFNKTPNVVQLLQVLFDTQAPLNAINKLPTVPMLGLT 900
                          ************** ****:***********************************:****

Med14Y                    QRSNTAYQCFSILPHSSTHIRLAFRNMYCIDIYCLSHGVVAVRDGAYSLCDNSKLVEGFC 957
Med14X                    QRTNTAYQCFSILPQSSTHIRLAFRNMYCIDIYCRSRGVVAIRDGAYSLFDNSKLVEGFY 960
                          **:***********:******************* *:****:******* *********

Med14Y                    PAPGLKTFLNMFVD----AQKRALKEDDYPPSPIGGDRTDSLISRLQPPQQQPFPKQPGI 1013
Med14X                    PAPGLKTFLNMFVDSNQDARRRSVNEDDNPPSPIGGDMMDSLISQLQPPQQQPFPKQPGT 1020
                          **************    *::*:::*** ********  *****:**************

Med14Y                    SGAYPLTSSPTSYRSPVNQPPSMMHTQPPG------------------------------ 1043
Med14X                    SGAYPLTSPPASYHSTVNQSPSMMHTQSPGNLHAASSPSGALRAPSPASFVPTPPPSSHG 1080
                          ********.*:**:*.***.*******.**
```

Figure S9 Med14Y protein. Protein sequences coded by the mRNA of figure S8 as determined using ExPASy translate tool aligned using ClustalW. * indicated conserved amino acids, : functionally conserved, and . slightly conserved.

```
Med14Y          --------------ALDSSSPYTLMSSSGGS----PPVFVSRLSPATPLPGMSPANPSLH 1085
Med14X          ISIGPGASFASPHGALDPSSPYTMVSPSGRAGNWPGSPQVSGPSPATRLPGMSPANPSLH 1140
                              ***.*****::*.** :     .  **  **** ************

Med14Y          STVPDVFHSPRAGTSSQTMPTNMSPPRKLPQRSCAASVPTILTHSALNILLLPSPMPGLV 1145
Med14X          SPVPDVSHSPRAGTSSQTMPTNMPPPRKLPQRSWAASIPTILTHSALNILLLPSPTPGLV 1200
                *.**** ****************.********* ***:***************** ****

Med14Y          PGLAGSYLCSPLERFLGSVIMRRHLQRIIQQETLQLISSNEPGVIMFKTDALKCRVTLSP 1205
Med14X          PGLAGSYLCSPLERFLGSVIMRRHLQRIIQQETLQLINSNEPGVIMFKTDALKCRVALSP 1260
                *************************************.******************:***

Med14Y          QTNQTLQLKVTPENVGQWKPDELQVLEKFFETRVAGPPFKANTLIAFTKLLGAPTHILRD 1265
Med14X          KTNQTLQLKVTPENAGQWKPDELQVLEKFFETRVAGPPFKANTLIAFTKLLGAPTHILRD 1320
                :*************.*********************************************

Med14Y          CVHIMQLEVFPDQATQLKWNVQFCLTIPPSAPPIAAPGTPAVVLKSKMLFFLQLTQKTSV 1325
Med14X          CVHIMKLELFPDQATQLKWNVQFCLTIPPSAPPIAPPGTPAVVLKSKMLFFLQLTQKTSV 1380
                *****:**:**************************.************************

Med14Y          PPQEPVSIIVPIIYDMASGITQQADIPRQQNSSVAAPMMVSTILKRFAEMNPPRQGECTI 1385
Med14X          PPQEPVSIIVPIIYDMASGTTQQADIPRQQNSSVAAPMMVSNILKRFAEMNPPRQGECTI 1440
                ******************* *********************.******************

Med14Y          FAAVCDLMAHLTLPPGGCP 1404
Med14X          FAAVRDLMANLTLPPGGRP 1459
                **** ****:******* *
```

Figure S9 Med14Y protein. Protein sequences coded by the mRNA of figure S8 as determined using ExPASy translate tool aligned using ClustalW. * indicated conserved amino acids, : functionally conserved, and . slightly conserved.

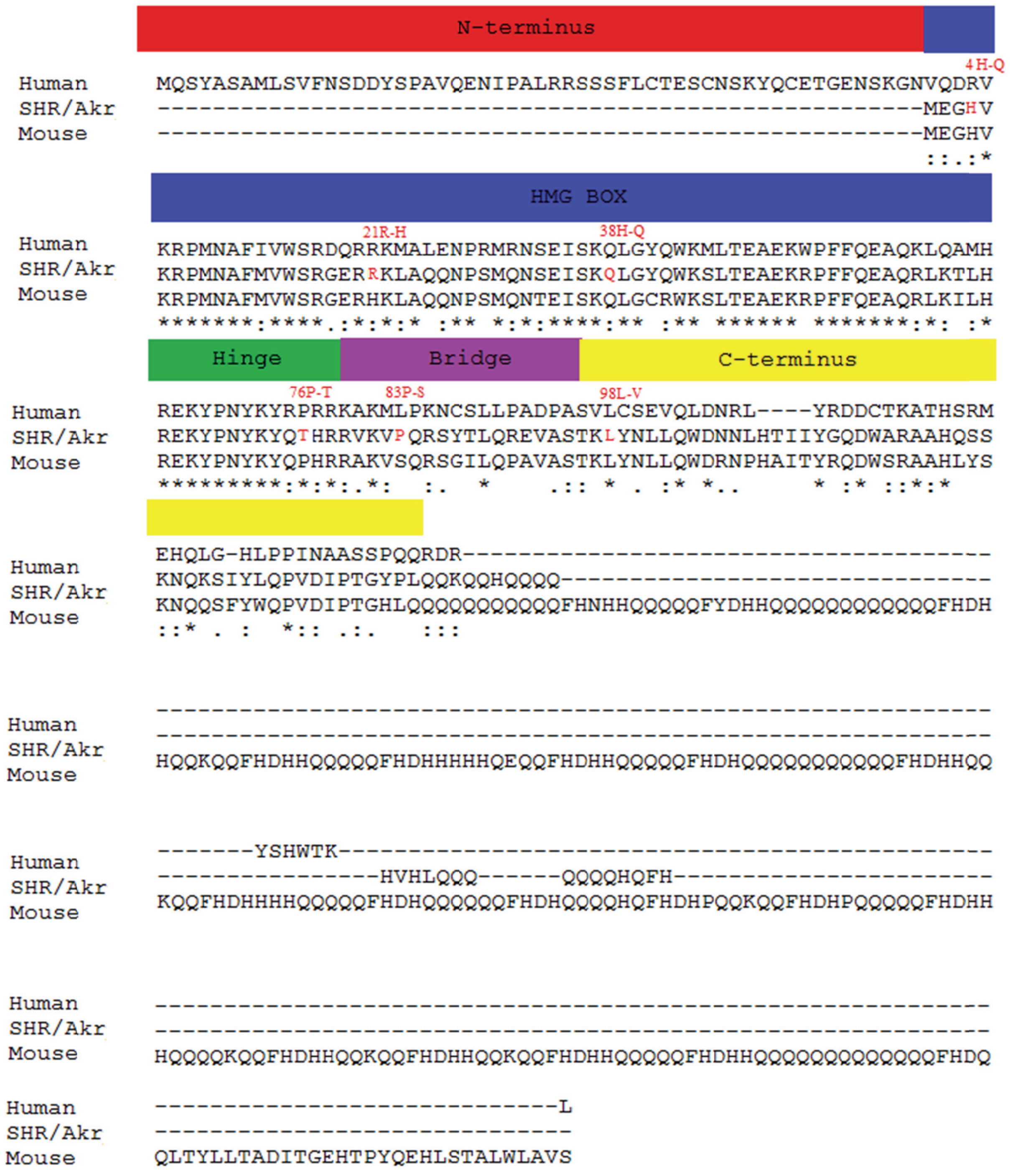

Figure S10 Human, Rat, and Mouse Sry protein alignments Sequence alignment of the rat Sry consensus from SHR/Akr aligned to Sry from mouse and human showing the location of the N-terminus (red), HMG box (blue), Hinge (green), bridge (magenta) and C-terminus (yellow). Amino acids differing in the multiple rat copies outside of the Q-rich region can be seen in red with the amino acid alternatives shown next to the number.

A

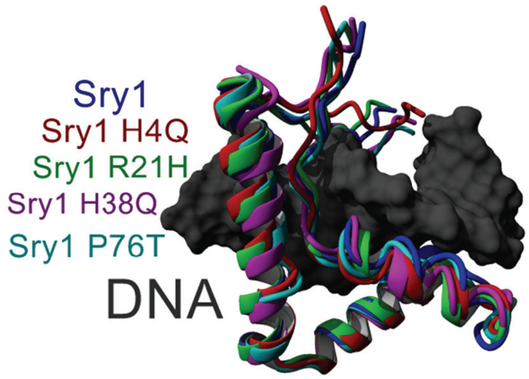

B

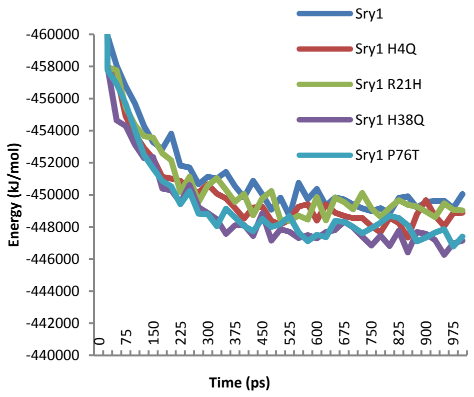

Figure S11 Mutations to the HMG box of Sry1 based on the multiple Sry copies. A). Energy minimized structures of the multiple models aligned together on DNA (gray). B). Energy of each model in **A** over the molecular dynamic simulation.

A

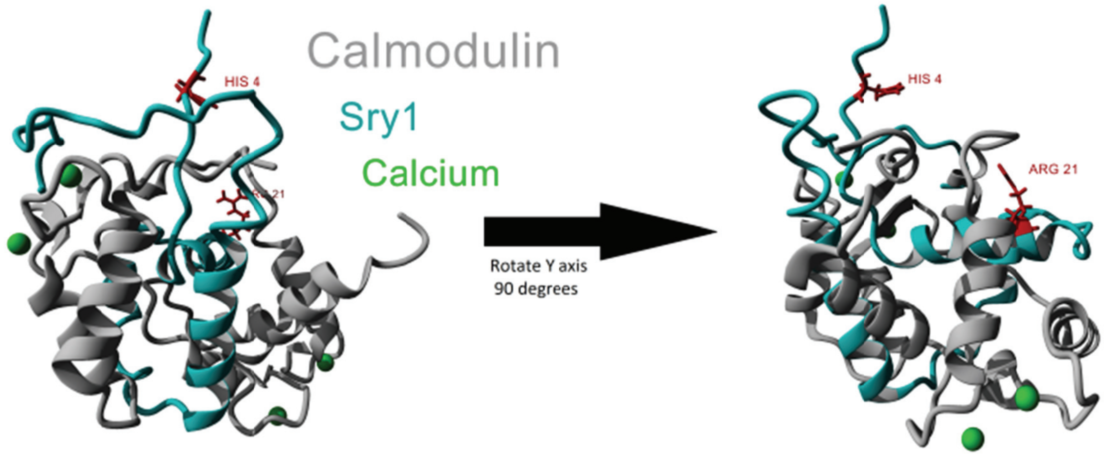

B

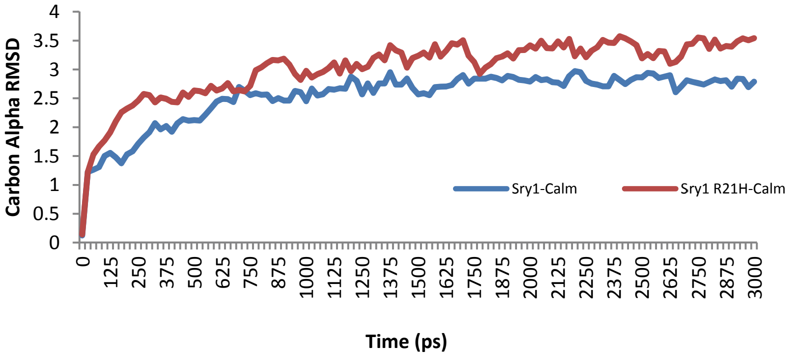

Figure S12 Role amino acid 21 change to interaction with Calmodulin. A). Modeled interaction between Sry and Calmodulin showing the location of amino acid 4 and 21 (red) both of which differ between all other rat Sry proteins and Sryβ. B). Molecular dynamic simulations of the modeled Sryα-Calmodulin interaction (A) and with amino acid 21 changed from an Arg to His leading to a decreased stability of the protein.
